# Networks in Aquatic Communities Collapse Upon Neonicotinoid‐Induced Stress

**DOI:** 10.1111/ele.70121

**Published:** 2025-04-22

**Authors:** S. Henrik Barmentlo, Maarten Schrama, Ellen Cieraad, Geert R. de Snoo, C. J. M. Musters, Peter M. van Bodegom, Martina G. Vijver

**Affiliations:** ^1^ Department of Environmental Biology, Institute of Environmental Sciences Leiden University Leiden the Netherlands; ^2^ Nelson Marlborough Institute of Technology Nelson New Zealand; ^3^ Netherlands Institute of Ecology (NIOO‐KNAW) Wageningen the Netherlands

**Keywords:** biodiversity, co‐occurrence, decomposition, ecosystem functioning, freshwater ecology, insecticide, invertebrates, pollution, primary production

## Abstract

Freshwater ecosystems worldwide are under pressure from neonicotinoid insecticides. While it is recognised that communities of species are responsible for ecosystem functioning, it remains unknown if neonicotinoid‐induced community transformations negatively affect ecosystem functioning. Therefore, we employed an experimental approach with 36 naturally established freshwater ecosystems exposed to increasing field‐realistic concentrations of the neonicotinoid thiacloprid. Upon exposure, we found severe degradation of ecosystem functioning in the form of loss of organic matter consumption and dramatic shifts in primary productivity. This functional decline coincides with strongly eroded species co‐occurrence networks to the point that these are indistinguishable from randomised assemblages of species. Together, these findings show how current environmental concentrations of a neonicotinoid can strongly disrupt freshwater ecosystem functioning via degradation of the invertebrate food web. Since this dramatic ecosystem degradation occurs below nearly all identified ecotoxicological risks, we call here for the reconsideration of the use of these insecticides.

## Graphical abstract

We employed an experimental approach with 36 naturally established freshwater ecosystems exposed to increasing field realistic concentrations of the neonicotinoid thiacloprid. Upon exposure, we found severe degradation of species co‐occurrence networks and coincident ecosystem functioning. Since this dramatic ecosystem degradation occurs below nearly all identified ecotoxicological risks, we call here for the reconsideration of the use of these insecticides.

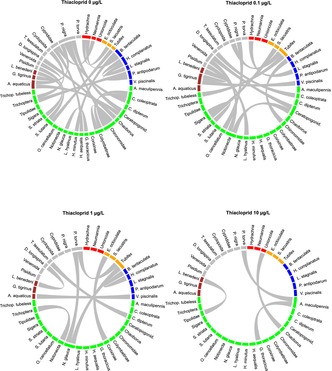

## 1 Introduction

We are currently witnessing an unprecedented decline in biodiversity (Díaz et al. 2019; Rockström et al. 2009; WWF 2018), which is generally attributed to anthropogenic impacts. Despite these global declines, species richness at the local community scale is often remarkably constant at decadal scales (Blowes et al. 2019; Dornelas et al. 2014) and sometimes even shows short‐term increases (Dornelas et al. 2014). This is widely attributed to species replacement due to colonising species from undisturbed adjacent habitats (Blowes et al. 2019). One anthropogenic disturbance that is suspected to impact natural communities and induce species replacement is the presence of neonicotinoid insecticides in freshwater ecosystems (Barmentlo, Schrama, et al. 2019; Schmidt et al. 2022; Stehle and Schulz 2015). Since the community of organisms locally present is responsible for the functioning of the local ecosystems (Hagan et al. 2021; Hooper et al. 2005), this begs the question: do neonicotinoid‐induced shifts in community composition result in a degradation of ecosystem functioning?

While there are few laws in ecology, at least a simple and perhaps trivial consensus is that in ecological systems ‘organisms interact with one another (…) and their environment’ (Lawton 1999). Hence, it may not be the species richness of a local community per se, but rather the set of functional links between organisms in a specific community and with their environment that provides the connection between biodiversity and ecosystem functioning (Hagan et al. 2021; Musters, Ieromina, et al. 2019; Valiente‐Banuet et al. 2015). Indeed, previous studies on the effects of disturbances (‘stress’, *sensu* Pickett et al. 1989) have shown that links between species and ecosystem process can be altered, consequently altering ecosystem functioning (Hooper et al. 2005). However, defining all possible ecological relationships and individually analysing how these respond to different stressors within a given ecosystem is close to impossible. This hampers the extent to which we can understand the effects of anthropogenic‐induced stresses to an ecosystem and its community.

Ecologists use network‐based analyses of species co‐occurrences to gain insight into the functional coherence of ecological communities (Barberán et al. 2012; Encinas‐Viso et al. 2016; Morriën et al. 2017; Ochoa‐Hueso et al. 2021; Simons et al. 2019). Such networks, when based on abundance measures, provide information on the statistical coherence (i.e., the number of correlations between species and correlation density, see also Neutel et al. 2002) of a given community (Ramirez et al. 2018). Although co‐occurrence networks have been evaluated theoretically and applied in field observations, building on concepts from the last decades (Berlow 1999; Hooper et al. 2005; Hutchinson 1959; Lawton 1999; Pimm et al. 1991), experimental evidence for the effectiveness of such network approaches in identifying the effect of anthropogenic stressors such as neonicotinoid exposure is currently lacking. We also lack understanding of the potential effects of neonicotinoid‐disrupted communities on ecosystem functioning. Neonicotinoids are a well‐known environmental stressor as they are used and found worldwide (Casado et al. 2019; Morrissey et al. 2015; Sánchez‐Bayo et al. 2016; Leiden University and Rijkswaterstaat‐WVL 2018) and are toxic to a wide range of individual invertebrate species (Barmentlo et al. 2021; Goulson 2013; Morrissey et al. 2015; Pisa et al. 2015; Vijver and van den Brink 2014; Woodcock et al. 2017). Neonicotinoids can also impede several freshwater ecosystem processes such as organic matter (‘OM’) decomposition, primary production or biomass transfer to neighbouring ecosystems (Barmentlo et al. 2021; Duchet et al. 2023; Sánchez‐Bayo et al. 2016). However, knowledge is lacking regarding to what extent neonicotinoid concentrations in surface waters actually affect natural freshwater biodiversity and how the functional coherence of the community is affected. To explore this gap, we analysed naturally assembled freshwater communities that were exposed to an environmentally realistic neonicotinoid concentration range by using a two‐tiered network‐based approach. First, we compared community co‐occurrence networks to randomly generated communities (i.e., a null model), and second, we evaluated how the neonicotinoid‐induced stress moves through the community and disrupts the concomitant ecosystem processes. We experimentally show that co‐occurrence network approaches can identify the effects of a neonicotinoid at very low concentrations and that co‐occurrence network disruption coincides with losses in ecosystem functioning.

## 2 Materials and Methods

### 2.1 Experimental Setup

**FIGURE 1:**
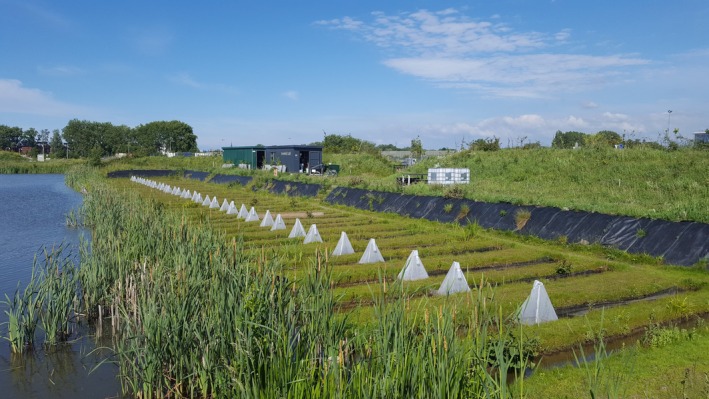
Overview of the experimental ditch system. Shown from left to right are the adjoining lake, wave barrier, helophyte layer (comprising of species such as *Typha latifolia* L. and *Mentha aquatica* L.), 1 cm mesh netting fish barrier, hydrological barrier, experimental ditch. Each ditch has a length of 10 m, a width at the surface level of 0.8 m and a depth of 0.3 m. Ditch banks are covered in organically grown mixtures of grass and clover species.

The experiment was conducted at the outdoor research facility ‘Living Lab’ of Leiden University, the Netherlands (see Supporting Information section ‘Study site and experimental overview’, and Barmentlo, Schrama, et al. 2019 and www.mesocosm.eu for an extensive site description). Our setup of 36 experimental freshwater ditch ecosystems (Figure 1) followed a ‘mesocosm’ design through ensuring that the ditches were similar in habitat structure and that the treatment was well‐replicated (*N* = 9). Our setup also allowed natural colonisation: We did not control how and which ecological invertebrate communities developed (see Supporting Information for details). This provided a controlled outdoor experimental design to study the effects of the neonicotinoid on naturally‐formed communities and their environment.

After 5 months of species colonisation, at the end of March 2018, the ditches were closed off hydrologically from the adjoining lake (see Figure 1, Supporting Information ‘Study site and experimental overview’ and Table S1). Starting on May 18th, the neonicotinoid thiacloprid was applied in two spikes (2 weeks apart, Table S1) of four environmentally relevant concentrations following a block design (0, 0.1, 1 and 10 μg/L). All data collection followed this same design to minimise or even out the effects of (1) potentially naturally occurring gradients, (2) time of sampling efforts on the collected data and (3) any meteorological disturbances. Technical details on neonicotinoid application and the environmental relevant range of thiacloprid can be found in the Supporting Information. Water samples (15 mL) were taken 5 cm below the water surface level 1 h after neonicotinoid application (to ensure homogenisation of the compound) and thereafter daily for 1 week and then twice per week. Thiacloprid concentrations were analysed using liquid chromatography–tandem mass spectrometry and degradation was modelled via first‐order kinetics (see Supporting Information and Barmentlo, Vriend, et al. 2019). Direct measurements after application of the two neonicotinoid spikes showed that our actual concentrations in the ditch were 0, 0.08, 0.85 and 9.86 μg/L. Thiacloprid had a short presence in water with DT50 of 3.6 days and DT90 of 12.0 days (see also Barmentlo et al. 2021).

### 2.2 Community Composition

Two‐and‐a‐half weeks before the first application of the neonicotinoid, we explored if natural colonisation had resulted in similar macroinvertebrate communities among the ditches (Table S1) by comparing communities following previous methodology (Barmentlo, Schrama, et al. 2019). In each ditch, a one‐meter compartment (i.e., 10% of the total ditch length) was temporally isolated from the remainder of the ditch by quickly placing two acrylic sheets over the ditch's width. Within this compartment, we caught all macroinvertebrates using dipping nets (150 μm) for the water column and sieves (500 μm) for the top layer (3–5 cm) of the sediment. We stopped sampling when subsequent nets and sieves were empty, i.e., when all macroinvertebrates had been caught. Invertebrates were then sorted by taxonomic order in Petri dishes and identified to the lowest possible taxonomic level using stereomicroscopes before being returned alive to their respective ditch. We reduced the total time of the identification effort by counting the homogenised subsets of highly abundant taxa (e.g., zooplankton) until at least 50 individuals or 25% of the whole sample had been counted. The whole procedure from catch to release took 1–2 h per ditch. Since macrofauna sampling is disruptive to the ditch ecosystem, we limited the sample to 10% of the whole ditch so that 90% would remain undisturbed. In June, 1 month after the first application, we again sampled and identified all invertebrates following the same method. We chose this 1 month time span after application since this produced notable and strong effects on community similarity in our earlier experiments (Barmentlo, Schrama, et al. 2019; Beentjes et al. 2022).

### 2.3 Statistics on Community Composition and Network

All statistical analyses were performed using R (version 3.5.1, R core team 2019). In order to assess if we caught all macroinvertebrate taxa within our ditch system (γ‐diversity), we built randomised species accumulation curves before and after application (pooled across all ditches) and also separately per treatment after application, using the function *specaccum* (999 permutations, package ‘*Vegan*’). We observed swift saturation of the number of identified taxa, indicating that the (near) full range of invertebrate species had been caught over the mesocosm system as a whole (Figure S9). Saturation of the treatment‐specific species accumulation curves was similar but returned fewer taxa per treatment (59–67 taxa per treatment) than all 36 ditches combined (85 taxa), indicating a treatment effect on species assembly (Figure S9).

We tested for effects of neonicotinoid concentration over time on various community metrics using separate linear mixed effect models (function *lme*, package ‘nlme’). Total abundance, abundance per taxonomic order, species richness and Shannon diversity (*H*; being the most used index) were the response variables, with neonicotinoid concentration (i.e., the four test concentrations) and time period (before and after neonicotinoid application) as explanatory variables. Ditch ID was a random factor to account for the repeated measures design.

Effects of thiacloprid treatment, time period and their interaction on community composition was assessed using Permutational multivariate analysis of variance (PERMANOVA, function *adonis2*, package ‘*Vegan*’, 999 permutations) with Ditch ID as a random factor. The interaction term treatment * time period represents the before‐after‐control‐impact (BACI) effect of thiacloprid. We analysed the data both using raw abundances and presence/absence in order to identify whether potential shifts in macroinvertebrate communities were due to shifts in relative abundances or because of species turnover. We used Bray‐Curtis as measure for dissimilarity for both the raw abundance and presence/absence data but also tested the Jaccard's index for presence/absence dataset as this is usually preferred. With highly similar results for both measures, we show Bray‐Curtis results to allow comparison with the raw abundance data. Time period was the strongest variable affecting the freshwater macroinvertebrate communities (*R* ^2^ = 0.34, *p* < 0.001), while the respective interaction of time period and thiacloprid was relatively weaker (*R* ^2^ = 0.04, *p* = 0.014). This is a common observation since the β‐diversity of freshwater macroinvertebrate communities is highly dynamic over time (Musters, Hunting, et al. 2019).

We also investigated if neonicotinoid disturbance led to community convergence (i.e., reduced beta dispersion) or divergence (i.e., increased beta dispersion) (Barmentlo, Schrama, et al. 2019), by assessing the homogeneity of multivariate dispersion using beta‐dispersion tests (function *betadisper*, package ‘*Vegan*’, 999 permutations). We found no significant effects (both April and June: *p* > 0.05).

We assessed community statistical coherence using the Spearman rank‐based correlation network analysis on the abundance data of the macroinvertebrates. This approach does not provide direct causal linkages between species. However, a statistical correlation between two species does provide an indication that there are one or more factors (such as other species or abiotic conditions) that might govern the abundance of both species (Blanchet et al. 2020; Ochoa‐Hueso et al. 2021). While this assumption does not always hold for individual co‐occurrences, since there can be other explanations that lead to observed co‐occurrences (Blanchet et al. 2020), the overall number and strength of all correlations relative to a control condition represent the statistical coherence of communities, removing the need to identify specific species‐species or biotic‐abiotic relationships (Morriën et al. 2017). The underlying assumption of this approach is that networks of co‐occurring species are, to some degree, structured and non‐random (Pimm 2002). Moreover, we assume that in undisturbed systems, there is a higher degree of statistical coherence compared with disturbed systems, and that an increase in disturbance results in lower coherence. Our results provide evidence for these assumptions, i.e., in undisturbed communities, the network was more connected than a random community, and that the statistical coherence decreases with increasing stress, compared to the random community. Prior to correlation analysis, we removed rare species (≤ 5 individuals per species per treatment) to avoid effects of random co‐occurrences. We selected a cutoff of 5 individuals as at this value a species occurred in at least over half of the replicates in each treatment. Differences between the number of correlations in the observed communities and in the randomised control community were assessed using chi‐square tests.

Next, we compared the correlation matrix of each treatment (neonicotinoid concentration) to a null model to identify potential effects of the treatment on biological organisation. The null models consisted of 1000 generated ‘communities’ with abundances randomly distributed across the observed taxa within the control treatment (> 5 individuals per species). Abundances were generated following a zero‐inflated Poisson distribution using the null probability (i.e., species non‐occurrences probability) and the mean total abundance of the nine control replicates.

More (stronger) correlations in an observed community than in a randomised community are a clear indication of statistical coherence. Fewer (stronger) correlations point towards a loss in biological organisation. To visualise and assess differences in co‐occurrences and network coherence between treatments, we used two different thresholds for |Spearman's *ρ*| to determine the number of correlations. We investigated the number of significant correlations (*p* < 0.05), represented by a |*ρ*| > 0.67; these are referred to as ‘significant correlations’. We also assessed correlations with |*ρ*| > 0.80, which we refer to as ‘strong correlations’. At even greater correlation strengths, there were too few correlations for a meaningful analysis and interpretation (e.g., only five correlations were present at |*ρ*| > 0.90). In the Results section, we present the figures for the strong correlations (i.e., |*ρ*| > 0.80), as at this correlation strength the co‐occurrence density allowed for easy interpretation, whereas the equivalent figures for the significant correlations (i.e., |*ρ*| > 0.67) can be found in the Figure S5. Finally, network ‘connectance’ (see Morriën et al. 2017) was calculated as a percentage by dividing the number of (both all significant and strong) correlations by all possible correlations of the control treatment. We investigated network connectance relative to the control, as this measure of connectance includes all possible deviations in connectance through both potential losses of possible correlations (due to, e.g., total abundance shifts) and losses in correlation strength.

### 2.4 Abiotic Conditions and Ecosystem Processes

We monitored several variables (temperature, pH, oxygen concentration, conductivity, turbidity) to assess the ditch ecosystems' abiotic state throughout the experiment. We quantified primary productivity by monthly assessments of biomass of larger primary producers (macrophytes, and floating algal beds ‘FLAB’) and weekly periphyton and phytoplankton assessments (as chlorophyll A). See Supporting Information for more details.

### 2.5 Functional Feeding Groups

Functional feeding group biomass is a stronger predictor of effects on ecosystem processes than its abundance‐based counterpart. Therefore, we converted all abundance data to biomass using the species‐specific length (in mm, see Supporting Information) and a mass–length relationship for aquatic invertebrates (Sabo et al. 2002) to estimate the mass per specimen (k, in mg):

$${\text{mass}}_{k}=0.11\times {\text{length}}_{k}^{1.79}$$

Information on the feeding preference trait modalities per species was obtained from www.freshwaterecology.info (accessed 6/3/2019) (Schmidt‐Kloiber and Hering 2015) (Table S2). Biomass per species was then multiplied by trait modalities to obtain the biomass per functional feeding group per ditch.

### 2.6 Statistics on Ecosystem Functioning

We analysed the possible effects of the neonicotinoid on the measured ecosystem processes using Piecewise Structural Equation Modelling (pSEM, package ‘*piecewiseSEM*’). We made two base assumptions on the effects of the neonicotinoid on the measured variables; (1) the neonicotinoid can only directly affect the macroinvertebrate functional feeding groups and consequently (2) all possible effects of the neonicotinoid on ecosystem processes must be through changes in the functional feeding groups. These assumptions are supported by literature: neonicotinoids are roughly a factor 10–10,000 more toxic to invertebrates than to algae or plants and at our test concentrations no plant or algae toxicity is expected (Raby et al. 2018). Our base model included four linear mixed effect models of all direct effects of the neonicotinoid (as actual average spike concentration) on the biomass of four different functional feeding groups and five models of direct effects of functional feeding group biomass on a specific ecosystem process (i.e., phytoplankton & Filter Feeder, periphyton & Grazer, FLAB & Shredder + Gatherer Collector (‘GC’), plant growth rates & Shredder + GC and macroinvertebrate OM consumption & Shredder + GC). For all models we used the block design as a random factor. For the potential effects on the three consumer feeding groups, we also included predator next to thiacloprid concentration as an explanatory variable to account for potential natural or cascading effects via this pathway. As one species can occur in multiple functional feeding groups (see Section 2.5, ‘Functional Feeding Groups’), we did not draw relationships between the different consumer groups. Next, we evaluated additional indirect effects of ecosystem processes that could potentially alter other ecosystem processes, such as the formation of FLAB that could affect the abundance of periphyton and phytoplankton due to competition for light and resources. These models were defined a priori. Model evaluation and comparison was performed using the Bayesian Information Criterion (BIC), χ^2^ and insignificance of the *p*‐value of the different models. Floating algal beds were generally not observed in the control or the low insecticide treatment (14/18 samples). Therefore, we included FLAB in the pSEM as either present or absent and used a general linear mixed effect model with a binomial distribution to explore the effects of GC + Shredder biomass on FLAB presence or absence.

## 3 Results

### 3.1 Colonisation of Experimental Ecosystems

To confirm that starting conditions were similar, we compared community metrics prior to neonicotinoid application. Invertebrate counts showed that the natural colonisation of the ditches was similar between the prospective treatments as indicated by the lack of significant differences in the number of individuals (1002 ± SE 12 individuals/m ditch), taxon richness (30.0 ± SE 0.1), β‐diversity (analysed via Bray‐Curtis index) and functional feeding groups (*p* > 0.05 for all comparisons, Supporting Information Results and Figures S1 and S2). Comparisons of other biotic (Chl. A, decomposition) and abiotic conditions (pH, O_2_, temperature, electrical conductivity, nitrate, phosphate, turbidity) between prospective treatments also found no significant differences (*p* > 0.05 for all variables, Table S3).

### 3.2 Neonicotinoid‐Driven Changes in Communities

**FIGURE 2:**
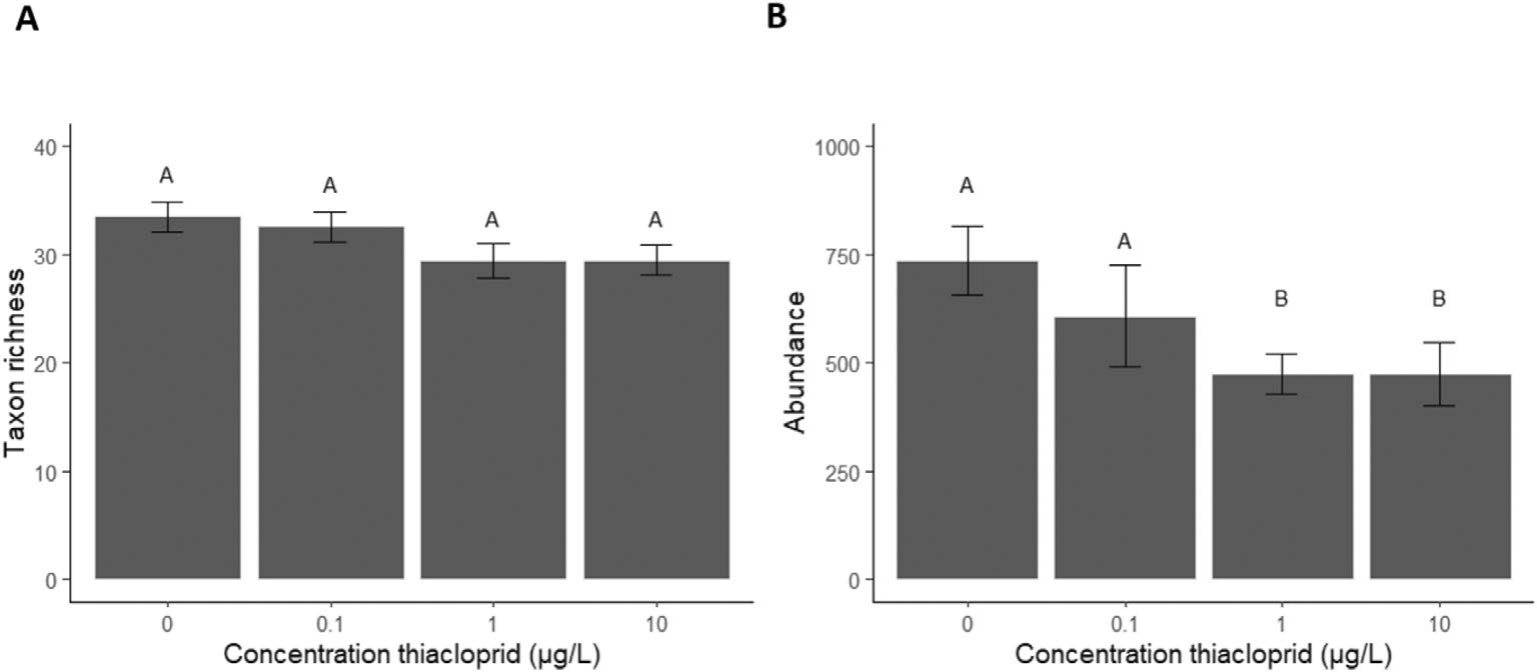
Average (*N* = 9, ±SEM) (A) Taxon richness and (B) total invertebrate abundance (per meter ditch) with increasing concentrations of thiacloprid. Different letters indicate differences at significance level *p* ≤ 0.05 (one‐way ANOVA with Dunnett's post hoc test).

One month after application, increasing neonicotinoid concentration had no significant effects on total taxon richness or Shannon diversity (One‐way ANOVA, *p* > 0.05; Figure 2A and Figure S3). However, total invertebrate abundance decreased by 18% at the lowest test concentration and by 36% at the two higher test concentrations (One‐way ANOVA, *F*_1,32_ = 4.6, *p* = 0.037; Figure 2B). This decrease in total abundance was mostly due to lower abundances in the taxonomic orders Malacostraca and Ostracoda (Table S4). In contrast, we observed higher abundances with increasing neonicotinoid concentration for the orders of Arachnida and Branchiopoda (Table S4), likely due to increased reproduction and/or offspring survival of these taxa with relatively short generation times.

Increasing thiacloprid concentrations induced increasingly significant dissimilarities in species composition compared to the control communities using either abundance (*R* ^2^ = 0.29, *p* < 0.001; Figure S4A) or presence‐absence data (*R* ^2^ = 0.24, *p* < 0.001; Figure S4B). This implies that the dissimilarity can be largely attributed to taxon replacement, which in turn explains the lack of effects on total taxon richness and Shannon diversity.

### 3.3 Disruption of the Invertebrate Network

**FIGURE 3:**
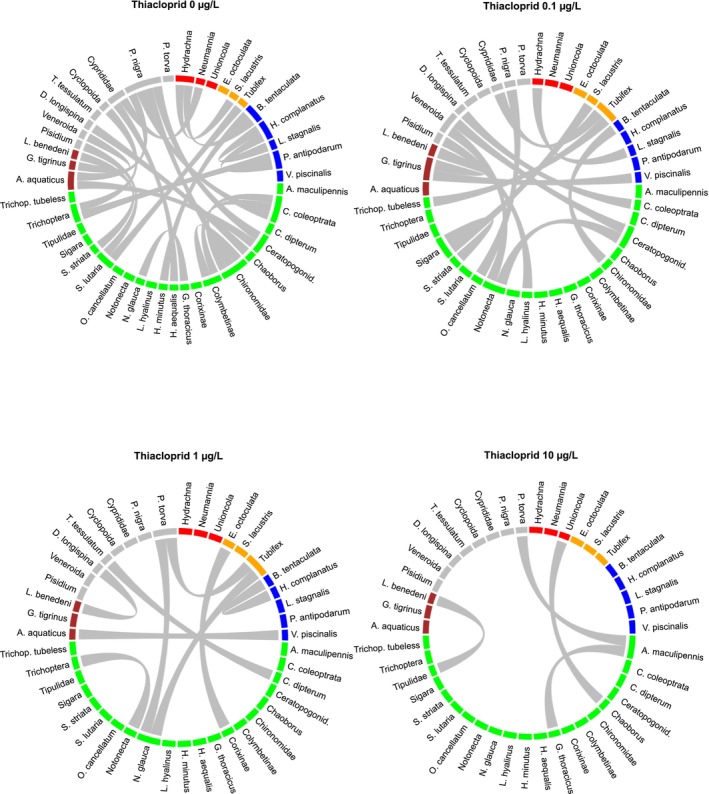
Effects of the neonicotinoid thiacloprid to aquatic invertebrate community co‐occurrence networks. Shown are the strong correlations with |Spearman's *ρ*| > 0.8 between different taxa per nominal spike concentration thiacloprid (0, 0.1, 1 and 10 μg/L). Green: Insecta, Orange: Clitellata, Blue: Gastropoda, Brown: Malacostraca, Red: Arachnida, Grey: Others. Note that the absence of a strong correlation does not mean the absence of the taxon as this taxon may be present in weaker correlations. The reduction of strong correlations with an increase in thiacloprid concentration coincided with both a reduction in total correlations (20%–36% relative to the control) as well as an overall decrease in correlation strength (median *R* ^2^ decreased from 0.60 in the control to 0.51 at 10 μg/L). The equivalent visualisation for all significant correlations (|Spearman's *ρ*| > 0.67) is presented in Figure S5.

Losses in invertebrate abundance also reduced the number of potential species‐species correlations (co‐occurrences) relative to the control by 8%, 30% and 33% for 0.1, 1 and 10 μg/L thiacloprid, respectively (Table 1 and Figure S5). The significant correlations (|Spearman's *ρ*| > 0.67) decreased (by 20%–36%) relative to the control (Table 1). The relative strength of these correlations also decreased, with median *R* ^2^ decreasing from 0.60 (control) to 0.55 (0.1 μg/L) to 0.53 (1 μg/L) to 0.51 (10 μg/L). This decrease in median *R* ^2^ aligned with a strong decline in the number of strong correlations (|Spearman's *ρ*| > 0.8): 23 strong correlations were observed within the control and this number decreased to 14, 9 and 4 correlations, respectively, with increasing insecticide concentrations (Figure 3). Since the total number of correlations was similar in the two highest test concentrations (43–40), these observed differences in losses between significant and strong correlations were probably due to shifts in taxa abundances and not due to species replacement (as also indicated by the different observed significant co‐occurrences, Figure S5). The remaining strong correlations within the neonicotinoid treatments were also different from those observed within the control (i.e., mostly different species co‐occurrences were found). In addition to the decline in the absolute number of correlations, there was also a decline in connectance (the correlations relative to the potential correlations in the control). This decline was similar for the significant correlations (|*ρ* > 0.67|) and for the strong correlations (|*ρ* > 0.80|) (Table 1).

**TABLE 1:** Network co‐occurrences based on species‐specific |Spearman's *ρ*| correlations.

Concentration thiacloprid (μg/L)	Number of correlations			Median *R* ^2^ of all sign. corr.	Connectance (%)	
	Potential correlations	Significant correlations (|*ρ*| > 0.67)	Strong correlations (|*ρ*| > 0.80)		Significant correlations (|*ρ*| > 0.67)	Strong correlations (|*ρ*| > 0.80)
0	934	62	23	0.60	6.64	2.46
0.1	852	49	14	0.55	5.25	1.50
1	626	43	9	0.53	4.60	0.96
10	619	40	4	0.51	4.29	0.43

*Note:* Significant correlations (*p* < 0.05) equate to a |Spearman's *ρ*| > 0.67, while strong correlations refer to those correlations with |Spearman's *ρ*| > 0.80. ‘Connectance’ represents the percentage of (significant or strong) correlations relative to the total number of potential correlations in the control (0 μg/L).

**FIGURE 4:**
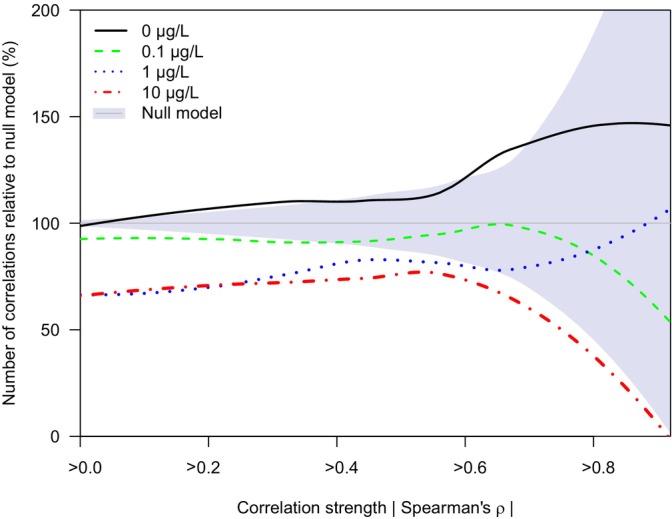
Relative number of co‐occurrences (versus the null model), per correlation strength (greater than or equal to |Spearman's *ρ*|) per thiacloprid concentration (using locally estimated scatterplot smoothing). The median number of correlations observed in the null model is set to 100% with the 0.025–0.975 quantiles shown in grey shading. The control treatment shows significantly more correlations than both the null model and the other three test concentrations, indicating that the control communities show biological organisation. In contrast, the number of co‐occurrences at thiacloprid concentrations 0.1, 1 and 10 μg/L was indistinguishable from the null model (*p* > 0.05), which shows the impairment of the ecological invertebrate networks. The equivalent visualisation showing the absolute number of co‐occurrences versus the null model is presented in Figure S6.

Control communities consistently showed more significant and stronger species' co‐occurrences than randomly generated communities (23% more correlations at |*ρ*| > 0.67, *p* < 0.05 and 74% more at |*ρ*| > 0.8, *p* < 0.05, Figure 4, Figure S6), which is a clear indication of non‐random structure of the control communities. We observed a clear loss in model‐estimated statistical coherence with insecticide application, as the neonicotinoid treatments 0.1, 1.0 and 10 μg/L showed no statistical difference (|*ρ*| > 0.67, *p* > 0.05), in terms of species' co‐occurrences relative to their generated random communities. At 10 μg/L, we even observed a strong reduction in the number of strong species' co‐occurrences (54% fewer correlations at |*ρ*| > 0.80, Figure 4), compared to the null model generated for the control community, i.e., a severe impairment of the statistical coherence at high neonicotinoid concentrations.

### 3.4 Loss of Ecosystem Functioning

**FIGURE 5:**
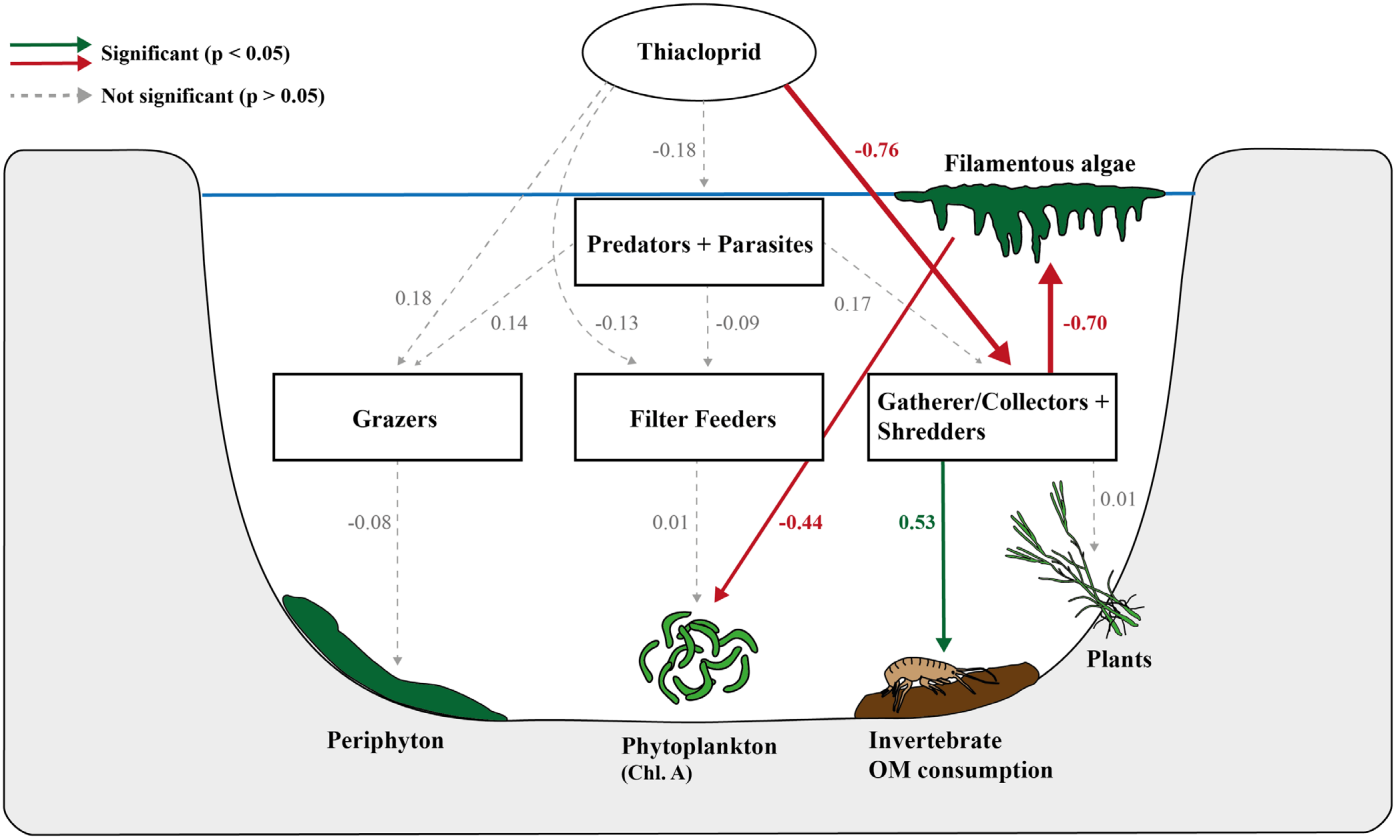
Piecewise Structural Equation Model (pSEM) of top‐down effects of increasing neonicotinoid insecticide (thiacloprid) concentrations on freshwater ecosystem functioning. Rectangular boxes indicate the different invertebrate functional feeding groups. Green and red arrows depict increasing and decreasing effects respectively and line width depicts the correlation strength (positive and negative standardised estimates for green and red arrows, respectively). Values for invertebrate organic material (OM) consumption and periphyton were derived from a 1 month growth rate, phytoplankton from a monthly time‐weighted average and plant and floating algal beds biomass from direct collection.

Insecticide‐induced stress might affect ecosystem functioning through many different direct and indirect pathways. We used a piece‐wise structural equation model (pSEM) to disentangle direct effects of the neonicotinoid on invertebrate functional feeding groups from indirect effects on ecosystem processes (organic matter consumption and primary productivity). The observed changes in statistical coherence was associated with coincident shifts in functional groups, as indicated by the pSEM. The biomass of the combined functional feeding group of ‘Gatherer/Collectors + Shredders’ decreased significantly by 15%, 46% and 85% for the insecticide concentrations 0.1, 1 and 10 μg/L, respectively, relative to the control (standardised regression coefficient estimate: −0.76, *p* < 0.001; Figure 5 and Figure S7). This coincided with changes in the ecosystem processes fulfilled by these functional feeding groups; invertebrate organic matter consumption strongly declined (standardised regression coefficient: 0.53, *p* < 0.001) by 80% and 100% at the 1 and 10 μg/L treatment relative to the control, respectively. Through losses in the ‘Gatherer/Collectors + Shredders’ functional feeding groups, ditches also became dominated by floating algal beds (standardised regression coefficient: −0.70, *p* = 0.012; Figure 5) with increases in biomass relative to the control of over 300 and over 1400% at 1 and 10 μg/L, respectively. Phytoplankton levels (as chlorophyll A concentration) also declined (9% and 23% at concentrations 1 and 10 μg/L, respectively) due to the increasing presence of FLABs (standardised regression coefficient: −0.44, *p* = 0.008) but not due to changes within the filter feeders (*p* > 0.05, Figure 5). This was probably due to competition for light and/or heat as the FLABs covered most of the water surface level (see Figure S8). We observed no significant decline in ecosystem functionality at the lowest test concentration. There were no significant changes in the biomass of other consumer feeding groups, nor in the ecosystem processes they are linked to.

## 4 Discussion

Our study uses a unique well‐replicated setup with 36 experimental freshwater ecosystems to show that ecosystems can exhibit a dramatic decrease in functioning upon neonicotinoid (thiacloprid) exposure, independent of changes in local species richness. To understand how this is happening, we explored how species co‐occurrence networks changed in response to the neonicotinoid‐induced stress. These analyses show that increasing levels of disturbance lead to a progressive loss of the number and strength of species co‐occurrences within these communities, to the point that almost all strong species correlations are lost, and our freshwater ecosystems cannot be distinguished from randomly generated communities. This reduction in the statistical coherence of ecological communities, which we used as a proxy of the functional coherence of the communities (Morriën et al. 2017), suggests that there may be important repercussions in terms of ecosystem functioning following this degradation of our observed co‐occurrence networks. Indeed, the observed progressive loss of species correlations from the co‐occurrence networks coincided with increasingly degraded ecosystem functioning, mostly due to progressive losses in biomass of two invertebrate functional feeding groups: ‘Gatherer/Collectors’ and ‘Shredders’. Importantly, these impacts on community structure and ecosystem functioning were not apparent from simple metrics such as taxon richness or α‐diversity, as these metrics remained stable despite a clear turnover of taxa.

While previous studies showed the potential of the network approach to assess community vitality in various ecosystems (Baiser et al. 2016; Barberán et al. 2012; Encinas‐Viso et al. 2016; Morriën et al. 2017; Ochoa‐Hueso et al. 2021; Simons et al. 2019), here we demonstrate the potential of the relationship between this vitality and the impacts of current environmental concentrations of a neonicotinoid. Moreover, we provide a direct reference to a natural control, while observational field studies are limited in this sense since they generally lack the normalisation of confounding (a)biotic variables. We found that co‐occurrence networks allowed identifying disturbances within a community despite intrinsic (stressor‐induced) differences in community composition. As our study shows, site‐specific null models based on the control state can evaluate the statistical coherence of each network. This approach allows early screening of (anthropogenically) disturbed communities, potentially even before any degradation in ecosystem functioning has taken place.

Using this network approach, our results show that current environmentally relevant levels of a neonicotinoid insecticide induce major compositional shifts and degradation of the aquatic food web shortly (i.e., 1 month) after pulse applications. These effects are well beyond any effects that could be estimated from species‐specific effects (as obtained from bioassays) described by standard risk assessment methodology (e.g., OECD 235, 2011; OECD 211, 2012). In addition, we already observed significantly impaired statistical coherence of invertebrate communities at nominal thiacloprid concentrations as low as 0.1 μg/L (actual 0.08 μg/L), which lies well below nearly all recognised toxic concentrations for individual aquatic species (Morrissey et al. 2015; Pisa et al. 2015; Raby et al. 2018). Furthermore, this concentration is close to, but below (1) a chronic 5% hazardous concentration of 0.146 μg/L for neonicotinoid compounds (Morrissey et al. 2015), (2) below the Environmental Protection Agency chronic benchmark for freshwater invertebrates of 0.97 μg/L; EPA 2024) and (3) similar to the EFSA Maximum Allowable Concentrations – Environmental Quality Standard (‘MAC‐EQS’) of 0.11 μg/L (Leiden University and Rijkswaterstaat‐WVL 2018). This indicates that these norms are insufficient for the protection of freshwater invertebrate communities. This also strongly suggests that the result of degrading co‐occurrence networks is not simply a result of differential thiacloprid induced mortality between species. Therefore, and because several taxa increased in abundance, the observed degradation is likely a result of both direct and indirect thiacloprid induced toxicity. The sensitivity of the co‐occurrence analysis of the ecological community shows that the current pesticide risk assessment has major shortcomings since it can fail to predict effects on the integrity of natural communities and related ecosystem functioning.

In our experiment, increasing environmentally relevant concentrations of thiacloprid resulted in the loss of the combined Shredder and Gatherer‐Collector functional feeding group, which were mostly dominated by non‐insects by a biomass ratio of 32:1 in the control and 6:1 for the highest test concentration. This loss, in turn, had pronounced consequences for ecosystem functionality both in terms of invertebrate consumption of detritus and filamentous algae beds, as well as cascading effects on primary production. Such changes in ecosystems are typically ascribed to effects of eutrophication, for example as a result of fertiliser runoff or leaching from a nearby agricultural field. As fertiliser and pesticides are generally applied simultaneously and found to jointly impair freshwater communities (Barmentlo, Schrama, et al. 2019), their effects may even interact with exacerbating impacts on ecosystem functioning (Birk et al. 2020). In addition to short‐lived impacts, recent research with thiacloprid at the same test facility showed that short‐term toxicity effects can lead to persistent restructuring of the invertebrate community (Barmentlo, Schrama, et al. 2019), which coincides with observations in long‐term studies on local community changes (Blowes et al. 2019; Dornelas et al. 2014). Because of this persistence, it is likely that the associated effects on ecosystem functioning found in our study also persist. While the use of thiacloprid in the European Union was discontinued in January 2020 (for a multitude of reasons, including suspected human carcinogenic properties; European Parliament 2019), neonicotinoid pesticides may still be used via emergency authorisation. In 2020 and 2021 this resulted in, as an example for sugar beets, 11 member states granting 17 authorisations for the use of several neonicotinoids. Overall, our results strongly support further, and increased, discontinuation of the use of neonicotinoid insecticides to preserve freshwater life and functioning.

In summary, our results show how a chemical pollutant, in the form of a neonicotinoid insecticide, can result in severe degradation of ecological co‐occurrence networks and ecosystem functioning. This is associated with changes in taxa composition but independent of changes in taxon richness. These results underscore the potential of co‐occurrence network analyses to identify impacts from anthropogenic disturbances on the functional coherence of communities. Our results also suggest an immediate re‐evaluation of the extensive usage of these insecticides is warranted, because both EPA and EFSA thresholds (that are deemed safe for freshwater life) were within our tested environmentally relevant concentrations where we observed degradation of freshwater communities and ecosystem functionality. Importantly, given the hundreds of pesticides that are on the market worldwide and present in the freshwater environment, the ecological networks and ecosystem functioning may be in far more perilous state than we have concluded based on trends in local taxon richness alone. We believe that our approach of linking experimentally controlled and naturally realistic network analysis to changes in ecosystem functioning allows for novel and comprehensive assessments to estimate the risks of anthropogenic stressors across ecosystems.

## Author Contributions

S.H.B., M.S., G.R.d.S., P.M.v.B. and M.G.V. conceptualised the manuscript. S.H.B. and M.S. collected the data. S.H.B., M.S. and E.C. analysed the data. All authors contributed to the writing of the manuscript.

## Conflicts of Interest

The authors declare no conflicts of interest.

### Peer Review

The peer review history for this article is available at https://www.webofscience.com/api/gateway/wos/peer‐review/10.1111/ele.70121.

## Supporting information


Data S1.

## Data Availability

All data and relevant codes can be obtained from the Dryad online data repository: https://doi.org/10.5061/dryad.b2rbnzsnk.

## References

[ele70121-bib-0001] Baiser, B. , R. Elhesha , and T. Kahveci . 2016. “Motifs in the Assembly of Food Web Networks.” Oikos 125: 480–491.

[ele70121-bib-0002] Barberán, A. , S. T. Bates , E. O. Casamayor , and N. Fierer . 2012. “Using Network Analysis to Explore Co‐Occurrence Patterns in Soil Microbial Communities.” ISME Journal 6: 343–351. 21900968 10.1038/ismej.2011.119 PMC3260507

[ele70121-bib-0003] Barmentlo, S. H. , M. Schrama , G. R. De Snoo , P. M. Van Bodegom , A. Nieuwenhuijzen , and M. G. Vijver . 2021. “Experimental Evidence for Neonicotinoid Driven Decline in Aquatic Emerging Insects.” Proceedings of the National Academy of Sciences of the United States of America 118, no. 44: e2105692118. 10.1073/pnas.2105692118. 34697235 PMC8612350

[ele70121-bib-0004] Barmentlo, S. H. , M. Schrama , P. M. van Bodegom , G. R. de Snoo , C. J. M. Musters , and M. G. Vijver . 2019. “Neonicotinoids and Fertilizers Jointly Structure Naturally Assembled Freshwater Macroinvertebrate Communities.” Science of the Total Environment 691: 36–44. 31306875 10.1016/j.scitotenv.2019.07.110

[ele70121-bib-0005] Barmentlo, S. H. , L. M. Vriend , R. H. A. van Grunsven , and M. G. Vijver . 2019. “Environmental Levels of Neonicotinoids Reduce Prey Consumption, Mobility and Emergence of the Damselfly *Ischnura elegans* .” Journal of Applied Ecology 56: 2034–2044.

[ele70121-bib-0006] Beentjes, K. K. , S. H. Barmentlo , E. Cieraad , et al. 2022. “Environmental DNA Metabarcoding Reveals Comparable Responses to Agricultural Stressors on Different Trophic Levels of a Freshwater Community.” Molecular Ecology 31: 1430–1443. 34908199 10.1111/mec.16326 PMC9306904

[ele70121-bib-0007] Berlow, E. L. 1999. “Strong Effects of Weak Interactions in Ecological Communities.” Nature 398, no. 6725: 330–334. 10.1038/18672.

[ele70121-bib-0008] Birk, S. , D. Chapman , L. Carvalho , et al. 2020. “Impacts of Multiple Stressors on Freshwater Biota Across Spatial Scales and Ecosystems.” Nature Ecology & Evolution 4: 1060–1068. 32541802 10.1038/s41559-020-1216-4

[ele70121-bib-0009] Blanchet, F. G. , K. Cazelles , and D. Gravel . 2020. “Co‐Occurrence Is Not Evidence of Ecological Interactions.” Ecology Letters 23: 1050–1063. 32429003 10.1111/ele.13525

[ele70121-bib-0010] Blowes, S. A. , S. R. Supp , L. H. Antão , et al. 2019. “The Geography of Biodiversity Change in Marine and Terrestrial Assemblages.” Science (New York, N.Y.) 366, no. 6463: 339–345. 10.1126/science.aaw1620. 31624208

[ele70121-bib-0011] Casado, J. , K. Brigden , D. Santillo , and P. Johnston . 2019. “Screening of Pesticides and Veterinary Drugs in Small Streams in the European Union by Liquid Chromatography High Resolution Mass Spectrometry.” Science of the Total Environment 670: 1204–1225. 31018436 10.1016/j.scitotenv.2019.03.207

[ele70121-bib-0012] Díaz, S. , J. Settele , E. Brondízio , et al. 2019. Summary for Policymakers of the Global Assessment Report on Biodiversity and Ecosystem Services of the Intergovernmental Science‐Policy Platform on Biodiversity and Ecosystem Services. IPBES secretariat.

[ele70121-bib-0013] Dornelas, M. , N. J. Gotelli , B. McGill , et al. 2014. “Assemblage Time Series Reveal Biodiversity Change but Not Systematic Loss.” Science (1979) 344, no. 6181: 296–299. 10.1126/science.1248484. 24744374

[ele70121-bib-0014] Duchet, C. , F. Hou , C. A. Sinclair , et al. 2023. “Neonicotinoid Mixture Alters Trophic Interactions in a Freshwater Aquatic Invertebrate Community.” Science of the Total Environment 897: 165419. 10.1016/j.scitotenv.2023.165419. 37429477

[ele70121-bib-0015] Encinas‐Viso, F. , D. Alonso , J. N. Klironomos , R. S. Etienne , and E. R. Chang . 2016. “Plant‐Mycorrhizal Fungus Co‐Occurrence Network Lacks Substantial Structure.” Oikos 125, no. 4: 457–467. 10.1111/oik.02667.

[ele70121-bib-0016] Environmental Protection Agency (EPA) . 2024. “Aquatic Life Benchmarks and Ecological Risk Assessments for Registered Pesticides.” https://www.epa.gov/pesticide‐science‐and‐assessing‐pesticide‐risks/aquatic‐life‐benchmarks‐and‐ecological‐risk.

[ele70121-bib-0017] European Parliament . 2019. P8_TA(2019)0199. Active Substances, Including Thiacloprid. European Parliament Resolution of 13 March 2019 on the Draft Commission Implementing Regulation Amending Implementing Regulation (EU) No 540/2011 .

[ele70121-bib-0018] Goulson, D. 2013. “An Overview of the Environmental Risks Posed by Neonicotinoid Insecticides.” Journal of Applied Ecology 50: 977–987.

[ele70121-bib-0019] Hagan, J. G. , B. Vanschoenwinkel , and L. Gamfeldt . 2021. “We Should Not Necessarily Expect Positive Relationships Between Biodiversity and Ecosystem Functioning in Observational Field Data.” Ecology Letters 24: 2537–2548. 34532926 10.1111/ele.13874

[ele70121-bib-0020] Hooper, D. , F. Chapin , J. Ewel , et al. 2005. “Effects of Biodiversity on Ecosystem Functioning: A Consensus of Current Knowledge.” Ecological Monographs 75, no. 1: 3–35. 10.1890/04-0922.

[ele70121-bib-0021] Hutchinson, G. 1959. “Homage to Santa Rosalia or Why Are There So Many Kinds of Animals?” American Naturalist 93, no. 870: 145–159. 10.1086/282070.

[ele70121-bib-0022] Lawton, J. H. 1999. “Are There General Laws in Ecology?” Oikos 84, no. 2: 177–192. 10.2307/3546712.

[ele70121-bib-0023] Leiden University (CML) , and Rijkswaterstaat‐WVL . 2018. Pesticide Atlas, Version 2.0 . www.bestrijdingsmiddelenatlas.nl.

[ele70121-bib-0024] Morriën, E. , S. E. Hannula , L. B. Snoek , et al. 2017. “Soil Networks Become More Connected and Take Up More Carbon as Nature Restoration Progresses.” Nature Communications 8, no. 14349: 1–10. 10.1038/ncomms14349 PMC5309817 28176768

[ele70121-bib-0025] Morrissey, C. A. , P. Mineau , J. H. Devries , et al. 2015. “Neonicotinoid Contamination of Global Surface Waters and Associated Risk to Aquatic Invertebrates: A Review.” Environment International 74: 291–303. 25454246 10.1016/j.envint.2014.10.024

[ele70121-bib-0026] Musters, C. J. M. , E. R. Hunting , M. Schrama , et al. 2019. “Spatial and Temporal Homogenisation of Freshwater Macrofaunal Communities in Ditches.” Freshwater Biology 64: 2260–2268.

[ele70121-bib-0027] Musters, C. J. M. , O. Ieromina , S. H. Barmentlo , et al. 2019. “Partitioning the Impact of Environmental Drivers and Species Interactions in Dynamic Aquatic Communities.” Ecosphere 10: e02910.

[ele70121-bib-0028] Neutel, A. M. , J. A. P. Heesterbeek , and P. C. De Ruiter . 2002. “Stability in Real Food Webs: Weak Links in Long Loops.” Science (1979) 296: 1120–1123. 10.1126/science.1068326 12004131

[ele70121-bib-0029] Ochoa‐Hueso, R. , M. Delgado‐Baquerizo , A. C. Risch , et al. 2021. “Ecosystem Coupling: A Unifying Framework to Understand the Functioning and Recovery of Ecosystems.” One Earth 4: 951–966.

[ele70121-bib-0030] Pickett, S. T. A. , J. Kolasa , J. J. Armesto , and S. L. Collins . 1989. “The Ecological Concept of Disturbance and Its Expression at Various Hierarchical Levels.” Oikos 54, no. 2: 129–136. 10.2307/3565258.

[ele70121-bib-0031] Pimm, S. 2002. Food Webs. University of Chicago Press.

[ele70121-bib-0032] Pimm, S. , J. H. Lawton , and J. E. Cohen . 1991. “Food Web Patterns and Their Consequences.” Nature 350, no. 6320: 669–674. 10.1038/350669a0.

[ele70121-bib-0033] Pisa, L. W. , L. P. Belzunces , J. M. Bonmatin , et al. 2015. “Effects of Neonicotinoids and Fipronil on Non‐target Invertebrates.” Environmental Science and Pollution Research 22, no. 1: 68–102. 10.1007/s11356-014-3471-x. 25223353 PMC4284392

[ele70121-bib-0034] R Core Team . 2019. R: A Language and Environment for Statistical Computing. R Foundation for Statistical Computing. http://www.r‐project.org/.

[ele70121-bib-0035] Raby, M. , M. Nowierski , D. Perlov , et al. 2018. “Acute Toxicity of 6 Neonicotinoid Insecticides to Freshwater Invertebrates.” Environmental Toxicology and Chemistry 37: 1430–1445. 29336495 10.1002/etc.4088

[ele70121-bib-0036] Ramirez, K. S. , S. Geisen , E. Morriën , B. L. Snoek , and W. H. van der Putten . 2018. “Network Analyses Can Advance Above‐Belowground Ecology.” Trends in Plant Science 23: 759–768. 30072227 10.1016/j.tplants.2018.06.009

[ele70121-bib-0037] Rockström, J. , W. Steffen , K. Noone , et al. 2009. “Planetary Boundaries: Exploring the Safe Operating Space for Humanity.” Ecology and Society 14, no. 2: 472–475. 10.5751/ES-03180-140232.

[ele70121-bib-0038] Sabo, J. L. , J. L. Bastow , and M. E. Power . 2002. “Length–Mass Relationships for Adult Aquatic and Terrestrial Invertebrates in a California Watershed.” Journal of the North American Benthological Society 21, no. 2: 336–343. 10.2307/1468420.

[ele70121-bib-0039] Sánchez‐Bayo, F. , K. Goka , and D. Hayasaka . 2016. “Contamination of the Aquatic Environment With Neonicotinoids and Its Implication for Ecosystems.” Frontiers in Environmental Science 4: 71. 10.3389/fenvs.2016.00071.

[ele70121-bib-0040] Schmidt, T. S. , J. L. Miller , B. J. Mahler , et al. 2022. “Ecological Consequences of Neonicotinoid Mixtures in Streams.” Science Advances 8: 1–13. 10.1126/sciadv.abj8182 PMC9007503 35417236

[ele70121-bib-0041] Schmidt‐Kloiber, A. , and D. Hering . 2015. “www.freshwaterecology.info – An Online Tool That Unifies, Standardises and Codifies More Than 20,000 European Freshwater Organisms and Their Ecological Preferences.” Ecological Indicators 53: 271–282.

[ele70121-bib-0042] Simons, A. L. , R. Mazor , and S. Theroux . 2019. “Using Co‐Occurrence Network Topology in Assessing Ecological Stress in Benthic Macroinvertebrate Communities.” Ecology and Evolution 9: 12789–12801. 31788214 10.1002/ece3.5751 PMC6875672

[ele70121-bib-0043] Stehle, S. , and R. Schulz . 2015. “Agricultural Insecticides Threaten Surface Waters at the Global Scale.” Proceedings of the National Academy of Sciences of the United States of America 112: 5750–5755. 25870271 10.1073/pnas.1500232112 PMC4426442

[ele70121-bib-0044] Valiente‐Banuet, A. , M. A. Aizen , J. M. Alcántara , et al. 2015. “Beyond Species Loss: The Extinction of Ecological Interactions in a Changing World.” Functional Ecology 29: 299–307.

[ele70121-bib-0045] Vijver, M. G. , and P. J. van den Brink . 2014. “Macro‐Invertebrate Decline in Surface Water Polluted With Imidacloprid: A Rebuttal and Some New Analyses.” PLoS One 9: e89837. 24587069 10.1371/journal.pone.0089837 PMC3938502

[ele70121-bib-0046] Woodcock, B. A. , J. M. Bullock , R. F. Shore , et al. 2017. “Country‐Specific Effects of Neonicotinoid Pesticides on Honey Bees and Wild Bees.” Science 356, no. 6345: 1393–1395. 10.1126/science.aaa1190. 28663502

[ele70121-bib-0047] WWF . 2018. Living Planet Report 2018: Aiming Higher. WWF.

