# Supplementary material for: Networks in Aquatic Communities Collapse Upon Neonicotinoid‐Induced Stress
Source: Ecol Lett. 2025 Apr 22;28(4):e70121. doi: 10.1111/ele.70121 (PMC12013465; doi:10.1111/ele.70121)
Supplement: Supplementary file 1 — Data S1. [file ELE-28-0-s001.docx]

**Supporting Information for**

Networks in aquatic communities collapse upon neonicotinoid-induced stress.

S. Henrik Barmentlo^1*^, Maarten Schrama^1^, Ellen Cieraad^1,2^, Geert R. de Snoo^1,3^, C.J.M. Musters^1^, Peter M. van Bodegom^1^, Martina G. Vijver^1^

^1^ Department of Environmental Biology, Institute of Environmental Sciences, Leiden University, PO Box 9518, 2300 RA Leiden, The Netherlands.

^2^ Nelson Marlborough Institute of Technology, Nelson, New Zealand.

^3^ Netherlands Institute of Ecology (NIOO-KNAW), Wageningen, the Netherlands.

**Corresponding author:** S. Henrik Barmentlo

**Email:** [s.h.barmentlo@cml.leidenuniv.nl](mailto:s.h.barmentlo@cml.leidenuniv.nl)

**Telephone:** +31-71-5277479

Methods

Study site and experimental overview

The site consists of 36 side-by-side experimental ditches, each 10m long, 0.8m wide at the surface level and 0.3m deep. Ditch banks are covered in an organically grown mixture of grass and clover. The ditches are connected to an adjacent water compensation level reservoir that provides a natural source of microbes, plants and invertebrates. After a 5-month colonization period, the ditches were isolated hydrologically by placing acrylic sheets (1000*500*2 mm) at the end of each ditch to prevent in- and outflow of the treatments (i.e., the neonicotinoid insecticide, see ‘Neonicotinoid application’). Since the ditches were only isolated hydrologically, colonization remained possible via aerial and terrestrial influxes and aquatic invertebrates are generally considered as fast colonizers (Cañedo-Argüelles & Rieradevall 2011). During sampling, to avoid edge effects, the 1 m section of each ditch we sampled at each sampling time was located near the middle of the 10 m long ditch. To avoid re-sampling a previously disturbed section of the ditch, the second sample was taken at 1 m distance from the previous sample site. The sampling compartment (left or right of the middle of the ditch) was randomized but the same for all ditches per period measured (3.5 - 4.5 m for the first and 5.5 - 6.5 m for the second sampling, respectively). The experimental period as a whole ran from early November until the end of June (35 weeks, Table S1). This includes an initial 5-month (22 weeks) colonization period before the ditches were hydrologically isolated. The ditches were isolated from the adjoining lake in the end of March (Table S1). Treatments were applied mid-May (see section ‘Neonicotinoid application and measurements’) and macroinvertebrate community and ecosystem functionality assessments were done 2-weeks before the first and 2-weeks after the second application of thiacloprid. After the first macroinvertebrate community assessment we allowed a recovery period of over 2 weeks since we found in our earlier experiments that within this time-frame large taxa shifts already naturally occur, meaning that fast reproducing taxa (such as zooplankton) were not exposed to this initial sampling stress (Beentjes *et al.* 2022).

Table S1.

**Table S1.** Overview of the activities and measurements over the course of the experiment. During the ‘colonization’, ditches are fully connected to the adjoining lake. Note that plant and floating algal beds were only collected during June since abundances were too low in April. Monthly measurements were performed in one month intervals before or after the first thiacloprid application (May 18^th^) and within the same month as close in time to each other as possible.

| **Month** | **Activity / measurement** |
| --- | --- |
|  |  |
| November | Colonization |
| December | Colonization |
| January | Colonization  Twice a month measurements of abiotic conditions and Chlorophyll A |
| February | Colonization  Twice a month measurements of abiotic conditions and Chlorophyll A |
| March | Colonization  Weekly measurements of abiotic conditions and Chlorophyll A. Monthly nutrient measurements.  Hydrological isolation of the experimental ditches (end of March). |
| April | Weekly measurements of abiotic conditions and Chlorophyll A. Organic matter consumption and decomposition over the course of the month.  Monthly nutrient measurements.  First macroinvertebrate sampling. |
| May | Treatment application (May 18^th^)  Weekly measurements of abiotic conditions and Chlorophyll A. Organic matter consumption and decomposition during the month.  Monthly nutrient measurements.  Thiacloprid measurements (daily during the first week after application and then twice per week).  Monthly periphyton built-up measurements. |
| June | Treatment application (June 1^st^)  Weekly measurements of abiotic conditions and Chlorophyll A. Decomposition over the whole month.  Monthly nutrient measurements.  Thiacloprid measurements.  Monthly plant sampling.  Monthly floating algal bed sampling.  Monthly periphyton built-up measurements.  Second macroinvertebrate sampling. |

Neonicotinoid application and measurements

Thiacloprid stock concentrations were prepared prior to spiking in demineralized water in 1L glass bottles. Thiacloprid was applied to the ditches in two separate but equal spikes, one on May 18^th^ (when neonicotinoid surface water concentrations are rising in the Netherlands and remain consistently high during the growing season (Barmentlo *et al.* 2018)) and one two weeks later (June 1^st^, Table S1), in four nominal concentrations; 0, 0.1, 1 and 10µg/L with nine replicate ditches per concentration. We aimed to maintain exposure to thiacloprid until the communities were sampled (see ‘Community structure’), which had been achieved using a two spike-design in an earlier study based on a measured half-life in water of 3.3 days (SD 0.1) (Barmentlo *et al.* 2019a). The range of neonicotinoid concentrations is considered environmentally relevant (Barmentlo *et al.* 2019b, 2021) for surface waters across the globe (Casado *et al.* 2019; Morrissey *et al.* 2015; Sánchez-Bayo *et al.* 2016; Stehle *et al.* 2018; Leiden University & Rijkswaterstaat-WVL 2018) as based on grab samples of surface waters. However note that due to high adsorption potential and the relative short half-lives in water, grab samples likely underestimate the peak concentrations (Barmentlo *et al.* 2019a). Stock concentrations of thiacloprid were diluted to the target concentration using 10L of filtered water (i.e. free of macroinvertebrates) from the ditch to which the spike was applied. This mixture was then spread evenly over the entire length of the ditch. We chose to administer thiacloprid via direct liquid application as this standard method of application allows for comparison with other ecotoxicological assessments. In addition, there are multitude of uses for thiacloprid and implementing all different exposure routes such as leaching and run-off within the experimental design is not feasible. Directly after sampling, samples were frozen at -20 ℃. Thiacloprid was measured using liquid chromatography-tandem mass spectrometry (Agent Technologies) following Roessink *et al.* (2013). The limit of detection was 0.012 ng/mL and the limit of quantification was 0.039 ng/mL.

Neonicotinoid effects on biodiversity metrics

For species richness, Shannon diversity and total macrofauna abundance, we observed no significant effects of the thiacloprid treatment in interaction with time period (p > 0.05 for all comparisons). However, Shannon diversity did differ significantly with time period, irrespective of treatment, from 1.7 before application to 2.3 after application on average (F1,34 = 108, p < 0.001), probably due to natural community development over the season. We also independently tested the response variables total macrofauna abundance, species richness and Shannon H per time period using one-way ANOVAs with Dunnett’s post-hoc tests to indicate differences between treatments after neonicotinoid application.

Abiotic conditions

To evaluate whether abiotic water conditions were (initially) similar, the standard water chemistry parameters temperature, pH, oxygen concentration, and conductivity were monitored on a weekly basis using a portable Hach multimeter (HQ 40d) and turbidity using a portable *Aqua*fluor® fluorometer. We started monitoring abiotic conditions in January to ensure that there was homogeneous water quality among mesocosms. In addition, nitrate and phosphate concentrations were measured from 15mL water samples collected 5cm below the surface water level in the middle of each ditch. These samples were collected and measured (using a NOVA 60 Spectroquant® photometer, Merck) one month before, 2h after and one month after the first neonicotinoid spike. The limit of detection for nitrate was 0.044mg/L and for phosphate 0.031 mg/L). None of these measured parameters showed statistical differences between treatments before or after application of the neonicotinoid, except for a decrease in turbidity at higher neonicotinoid concentrations (9% at 1 µg/L and 19% decrease at 10µg/L; F_1,34_ = 10.6, p=0.002; See Supp. Info. Table S3) after application. The time-weighted average turbidity was strongly correlated to chlorophyll A (see ‘Primary production’, two-tailed Pearson’s R^2^ = 0.80, p<0.001), and as such the declining turbidity could potentially be ascribed to the observed losses in phytoplankton (see ‘Loss of ecosystem functioning’). Additionally, decreasing turbidity may also be explained by losses in taxa of the ‘Gatherer Collector’ and ‘Shredder’ functional feeding groups, since several taxa belonging to these groups are active bioturbators and their loss likely resulted in less stirring of the sediment particles (as has also been observed by reference (Sánchez-Bayo & Goka 2006) and (Hayasaka *et al.* 2012)).

Species’ biomass and trait modalities

As we performed live invertebrate identifications, we used the maximal body length as described in identification literature as a proxy for biomass per species instead of determining dry weight. In some instances, individuals were too young to be identified to the species level. For these individuals, we set an arbitrary standard of 25% of the mean maximum length of the genus as derived from the identification literature. We selected 25%, since below this length it is often impossible to perform live identifications because the first instars do not have sufficient characteristics. For the few occurrences were this was the case (less than 1% of all sampled macrofauna), we only included species from literature that were actually identified to species level in the ditches to calculate this standardization.

The obtained functional feeding groups were: Miners, Xylophagous, Predator, Grazer, Filter Feeder (active and passive), Gatherer-Collector, Shredder, Parasite and Other (see Supp. Info. Table S2 for the food sources per functional feeding group). We did not identify miner or xylophagous species and therefore these functional feeding groups were omitted from further analyses. Importantly, a species can occur in multiple functional feeding groups due to shared feeding modes. This is reflected in the database as a fraction per functional feeding group (for instance: 0.5 shredder and 0.5 predator). For taxa that were not identified to the species level, we averaged the trait modality for all species within the respective taxonomic level. We summed the trait modalities of 1) ‘Predator’ and ‘Parasite’ and 2) ‘Active Filter Feeder’ and ‘Passive Filter Feeder’ as they filled similar niches within our ecosystem functioning model (for example: passive and active filter feeder both filter phytoplankton) and those of 3) ‘GC’ and ‘Shredder’ because they were strongly correlated since individual species often occurred in both functional feeding groups (i.e. ‘GC’ and ‘Shredder’; Pearson correlation: R^2^ = 0.95, p < 0.001).

Table S2.

**Table S2.** Food sources of the different functional feeding groups. Directly copied from the [www.freshwaterecology.info](http://www.freshwaterecology.info) database (accessed 8/12/2022)^54^.

| **Functional  feeding group** | **Explanation** |
| --- | --- |
| grazers | feed on endolithic and epilithic algal tissues, biofilm, partially POM, partially tissues of living plants |
| miners | feed on leaves of aquatic plants, algae and cells of aquatic plants |
| xylophagous taxa | feed on woody debris |
| shredders | feed on fallen leaves, plant tissue, CPOM |
| gatherers/collectors | feed on sedimented FPOM |
| active filter feeders | feed on suspended FPOM, CPOM; micro prey is whirled; food is actively filtered from the water column |
| passive filter feeders | feed on suspended FPOM, CPOM, prey; food is filtered from running water, e.g., by nets or specialized mouthparts |
| predators | feed on prey |
| parasites | feed on host |
| other feeding types | use other food sources not meeting the above categories |

Primary production

Macrophytes and floating algal beds were collected during the invertebrate community assay by sorting the samples from the community assessment into primary producer and invertebrate material. Samples were dried for three days at 40^0^C before determining dry weight per taxon. Monthly growth of periphyton before and after neonicotinoid application was determined as colonization of acrylic plates that had been inserted vertically in each ditch sediment for one month. Periphyton was scraped off the plates 1cm above the sediment level (to minimize the effect of sediment particles) and vacuum filtered onto a pre-weighed GF/C Whatmann filter which was subsequently dried at 40^0^C for three days before weighing again to determine periphyton dry weight. Phytoplankton abundance in each ditch was estimated using weekly chlorophyll A measurements with a handheld fluorometer (*Aqua*fluor®). The weekly data was used to calculate a time-weighted average for the month after the first thiacloprid spike.

Organic matter decomposition and consumption

The monthly rate of microbial decomposition and invertebrate consumption were measured by using DECOTABs were prepared by blending finely ground hay (30g) into an agar-agar solution (10g per 500mL demineralized water) which was allowed to set in cubicle silicon templates of 2*2cm. Each ditch then received a DECOTAB enclosure constructed of 2cm high round PVC circles (Ø 11cm, fitted with 0.5cm mesh to hold the DECOTABs in place) in the middle of the ditch (being close to the sampled invertebrate communities, see ‘community structure’). Each enclosure was fitted with two identically prepared DECOTABs except that one was covered with fine mesh (100µm) to exclude invertebrate consumption thus allowing for the measurement of microbial decomposition only. DECOTABs were retrieved after one month, carefully rinsed with water and then dried for 3 days at 40 ^0^C before weighing. Invertebrate consumption was inferred by subtracting microbial decomposition from the total degradation as derived from the DECOTAB without fine mesh.

Results

Fig. S1.


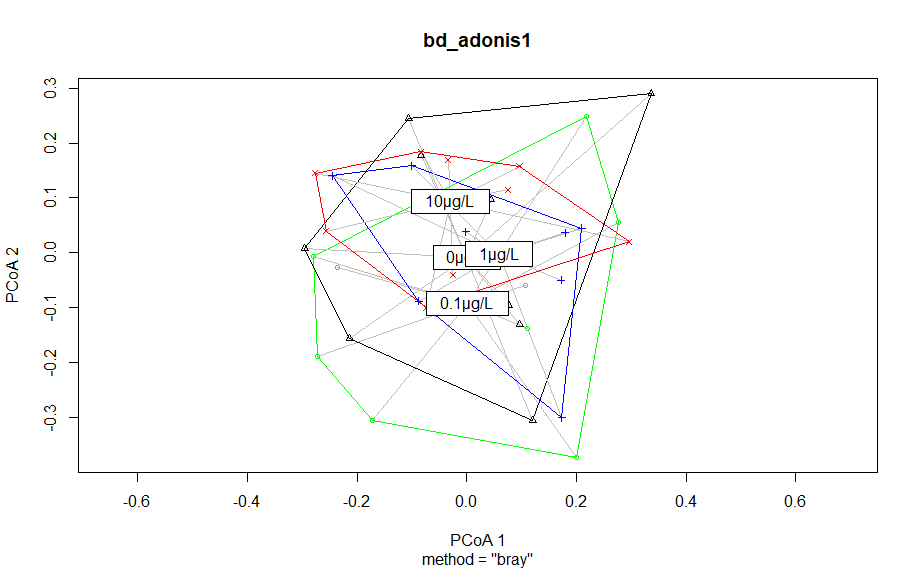


**Figure S1.** Principal coordinate analysis (PCoA; Bray-Curtis dissimilarity) of the prospective treatments (N = 9). Communities were sampled in April, one month before application of the neonicotinoid thiacloprid. Multivariate analyses (permanova and beta dispersion tests) showed no significant differences (p > 0.05) between the prospective treatments. Black: 0, green: 0.1, blue: 1 and red: 10µg/L thiacloprid).

Fig. S2.

**Figure S2.** Circular diagram of the different functional feeding groups of the prospective treatments one month before application of thiacloprid.

Table S3.

**Table S3.** Abiotic conditions before and after application of the neonicotinoid thiacloprid. Values are expressed as a monthly time-weighted average (N = 9) based on weekly measurements.

| **Parameter** | | **Neonicotinoid concentration (µg/L)** | | | |
| --- | --- | --- | --- | --- | --- |
|  |  | **0** | **0.1** | **1** | **10** |
| **Conductivity**  **(µS/cm)** | Before | 684 | 689 | 687 | 684 |
|  | After | 509 | 515 | 511 | 501 |
| **Nitrate** | Before | 0.34 | 0.31 | 0.82* | 0.31 |
| **(mg/L)** | After | 0.40 | 0.54 | 0.66 | 0.55 |
| **Oxygen**  **(mg/L)** | Before | 12.2 | 12.1 | 12.1 | 11.9 |
|  | After | 15.4 | 15.0 | 15.0 | 15.7 |
| **pH** | Before | 8.2 | 8.1 | 8.2 | 8.1 |
|  | After | 8.6 | 8.5 | 8.6 | 8.8 |
| **Phosphate** | Before | 0.12 | 0.20 | 0.15 | 0.20 |
| **(mg/L)** | After | 0.09 | 0.12 | 0.09 | 0.06 |
| **Temperature**  **(^0^C)** | Before | 17.8 | 17.8 | 17.8 | 17.7 |
|  | After | 23.9 | 23.8 | 23.9 | 23.9 |
| **Turbidity**  **(NTU)** | Before | 91.7 | 96.3 | 86.0 | 85.3 |
|  | After | 115.7 | 112.3 | 105.8 | 93.8 |

*strongly skewed by one outlier; the average concentration is 0.14mg/L (SE 0.07) when the outlier is excluded. Note that nitrate concentrations before thiacloprid application were often below the DL (0.04mg/L) and nutrients levels were thus oligotrophic.

Table S4.

**Table S4.** Average macroinvertebrate taxonomic class abundance (N = 9) one month after the first application of the neonicotinoid thiacloprid. One-way ANOVA results indicated no significant differences in abundance per treatment of all taxonomic orders before application of the neonicotinoid thiacloprid (p > 0.05 for all comparisons). Details of significant interaction effects on abundance per class of the neonicotinoid treatment and period (before and after application) are indicated (note that there were no significant effects of only the applied concentration).

| **Taxonomic Class** | **Neonicotinoid concentration (µg/L)** | | | | **Treatment * period interaction** |
| --- | --- | --- | --- | --- | --- |
|  | **0** | **0.1** | **1** | **10** |  |
| Clitellata | 6 | 9 | 6 | 5 | *NS* |
| Hirudinea | 8 | 3 | 9 | 5 | *NS* |
| Arachnida | 5 | 5 | 11 | 25 | F_1,34_ = 21, p < 0.001 |
| Branchiopoda | 88 | 58 | 66 | 153 | F_1,34_ = 5, p < 0.001 |
| Insecta | 112 | 126 | 89 | 75 | *NS* |
| Malacostraca | 255 | 214 | 139 | 34 | F_1,34_ = 18, p < 0.001 |
| Maxillopoda | 39 | 21 | 33 | 13 | *NS* |
| Ostracoda | 50 | 22 | 9 | 0 | F_1,32_ = 14, p < 0.001 |
| Bivalvia | 3 | 4 | 4 | 3 | *NS* |
| Gastropoda | 168 | 142 | 106 | 157 | *NS* |
| Rhabditophora | 2 | 3 | 2 | 3 | *NS* |

The order of Entognatha and Hydrozoa are not shown as abundances were generally lower than 1 individual per ditch. ‘NS’ = not significant.

Fig. S3.

**Figure S3.** Average (N = 9, ±SE) Shannon diversity (*H*) (per meter ditch) with increasing concentrations of thiacloprid one month after application. Different letters indicate differences (One-way ANOVA with Dunnett’s post hoc test) at significance level p < 0.05.

Fig. S4.

**A**


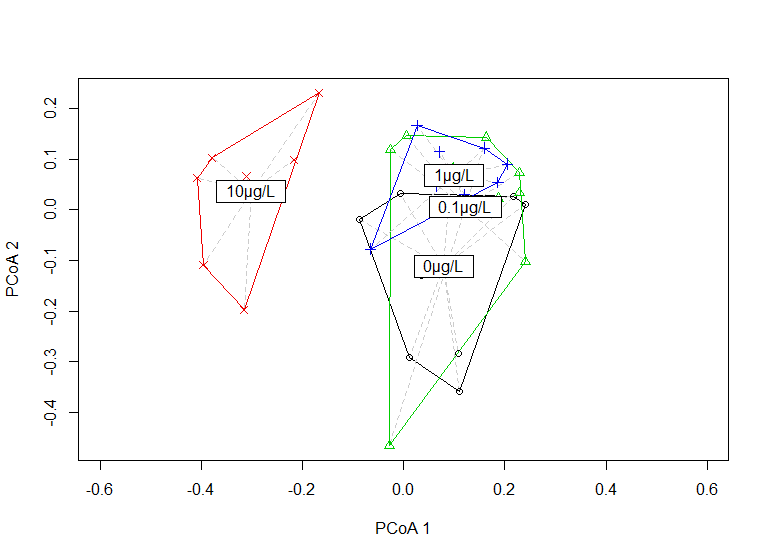


**B**


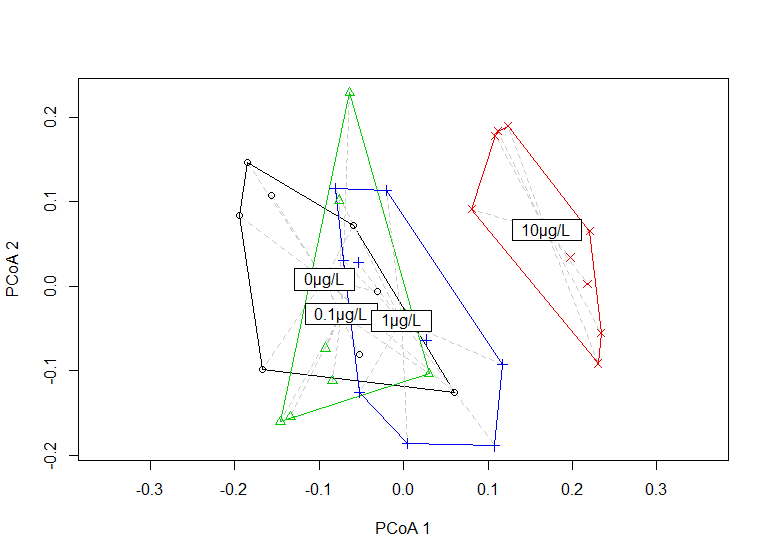


**Figure S4**. Principal coordinate analysis (PCoA; Bray-Curtis dissimilarity) of (A) raw abundance and (B) presence-absence data of freshwater invertebrate communities. Dissimilarity is show per nominal concentration of the neonicotinoid insecticide thiacloprid one month after application (N = 9; black: 0, green: 0.1, blue: 1 and red: 10µg/L).

Figure S5.


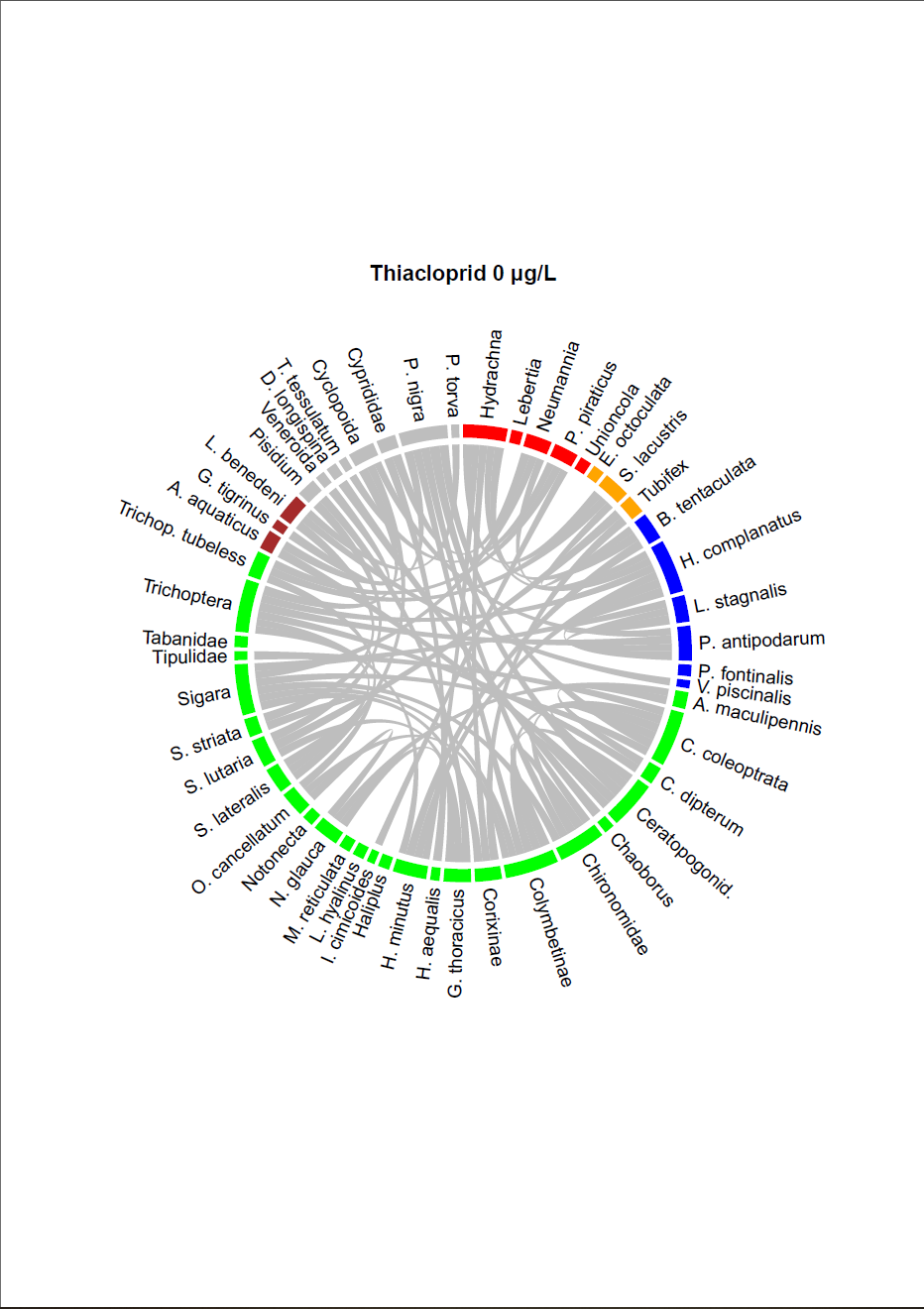

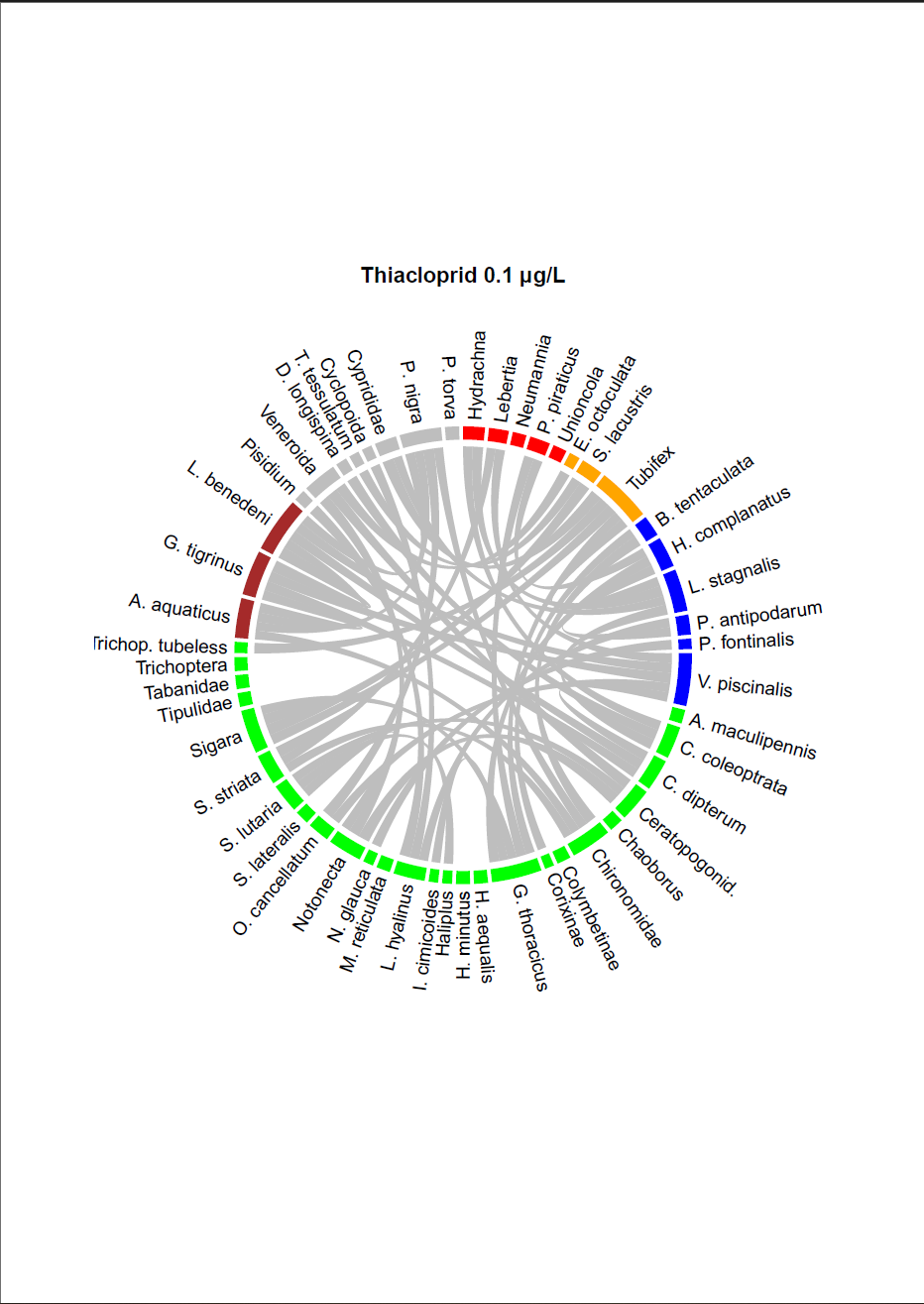


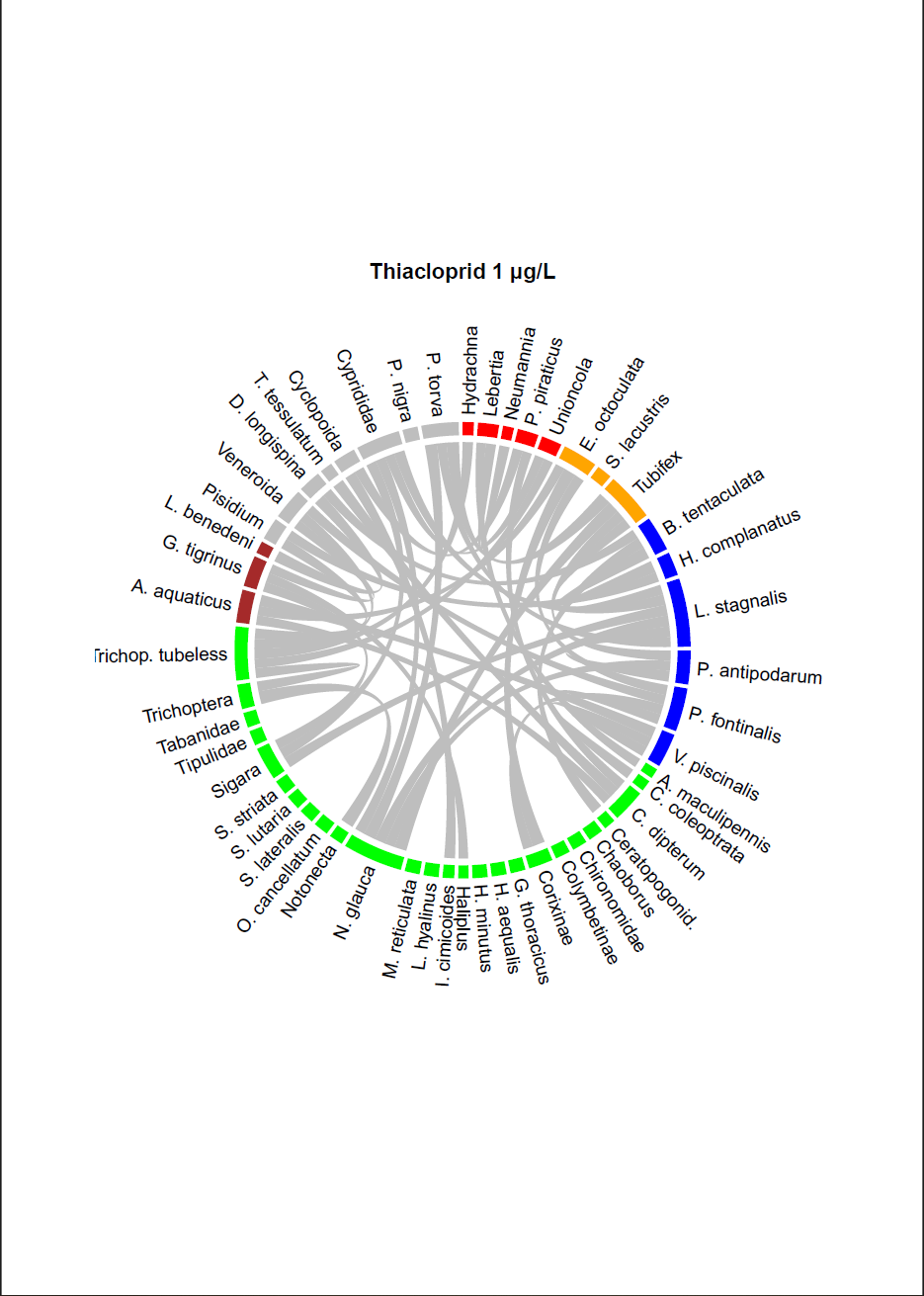

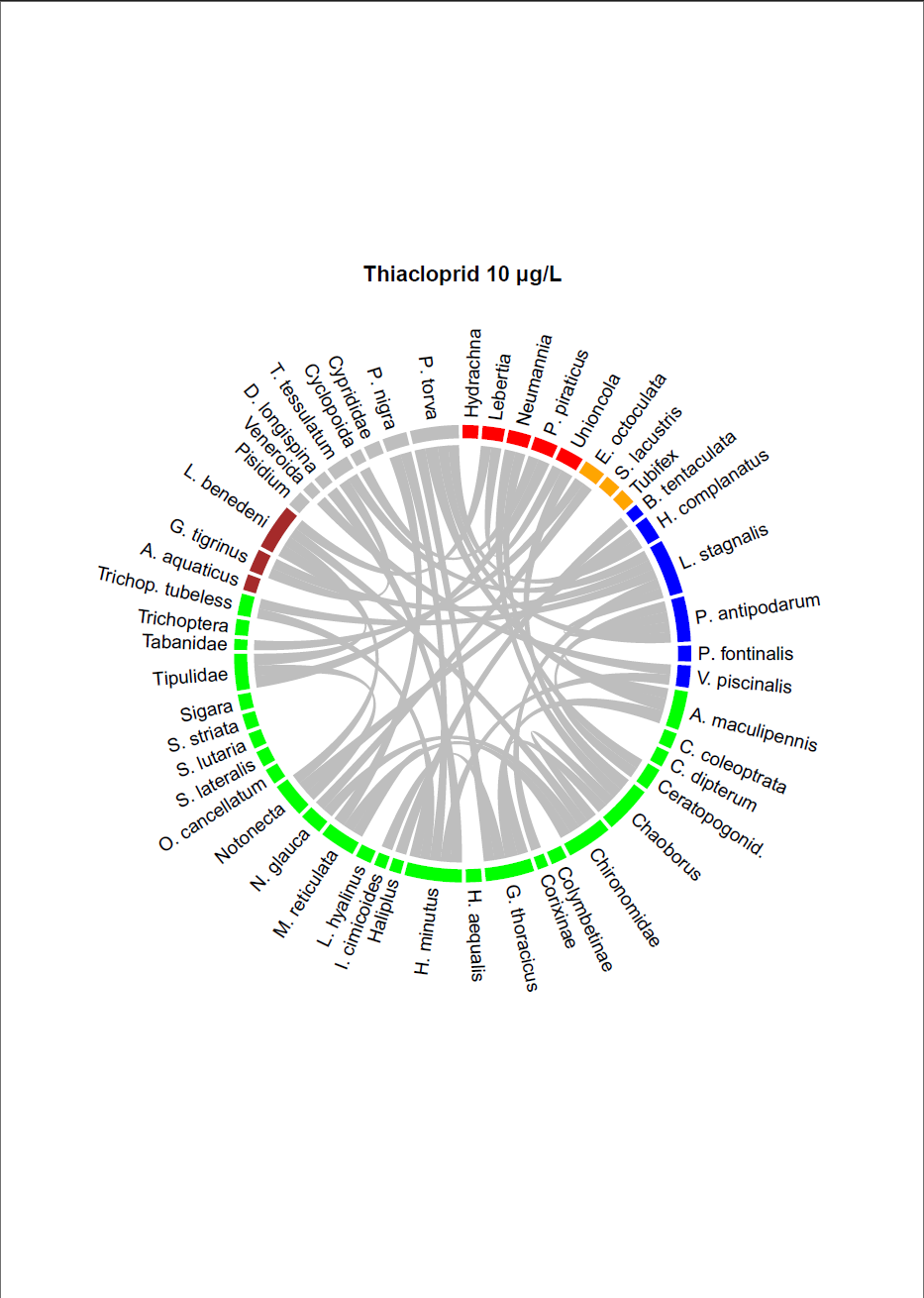


**Figure S5.** The effects of the neonicotinoid thiacloprid to aquatic invertebrate community co-occurrence networks. Shown are all significant correlations (| Spearman’s ρ | > 0.67, p < 0.05) between different taxa per spike concentration thiacloprid (0, 0.1, 1 and 10µg/L). Green: Insecta, Orange: Clitellata, Blue: Gastropoda, Brown: Malacostraca, Red: Arachnida, Grey: Others.

Fig. S6.


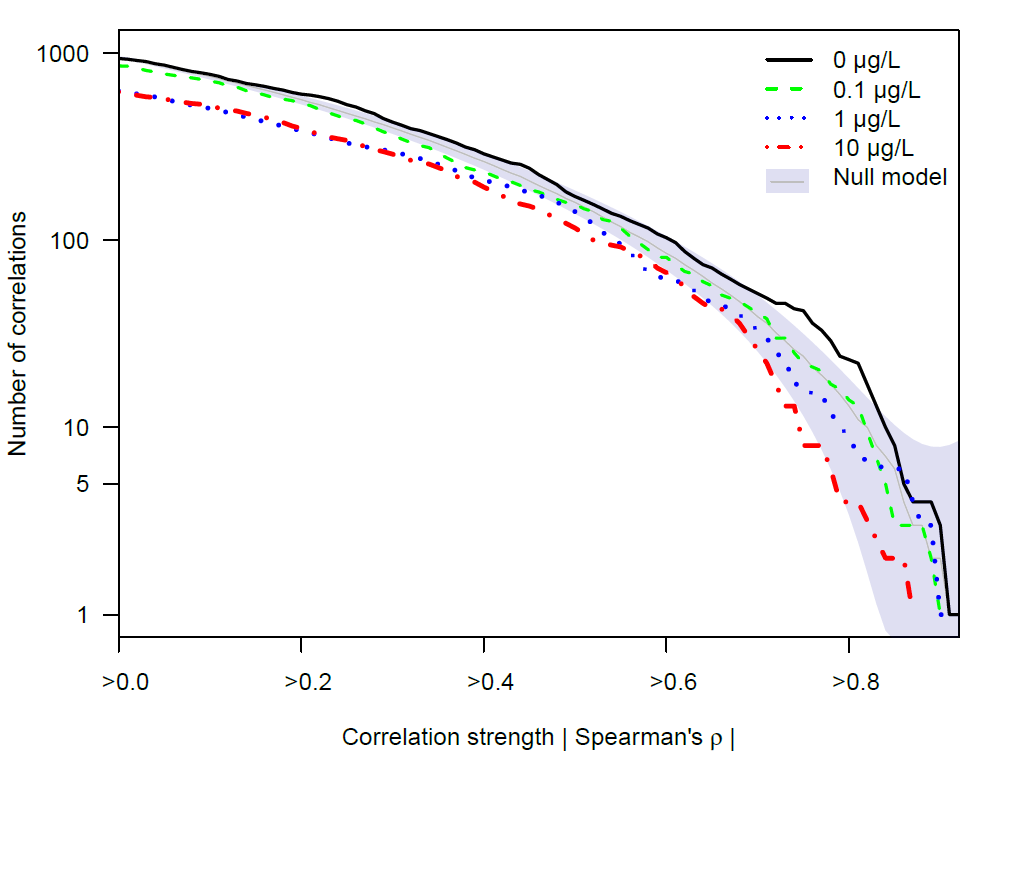
**Figure S6.** Cumulative number of co-occurrences (versus the null model), per correlation strength (greater than or equal to | Spearman’s ρ |) per thiacloprid concentration. The median number of correlations observed in the null model are set to 100% with the 0.025-0.975 quantiles shown in grey shading.

Fig. S7.

**
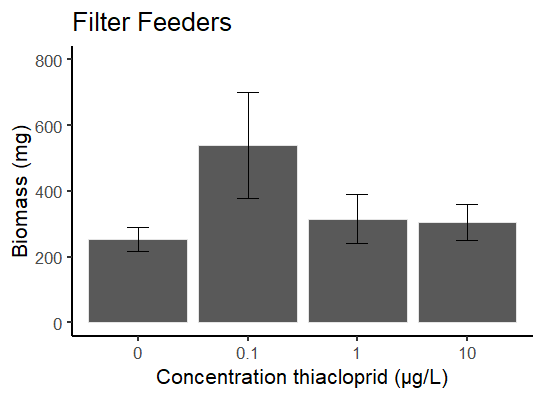

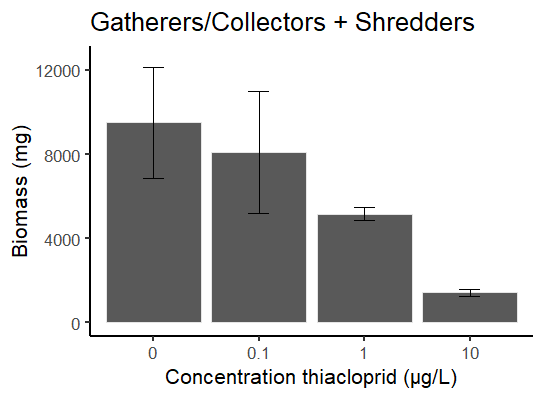
**

**
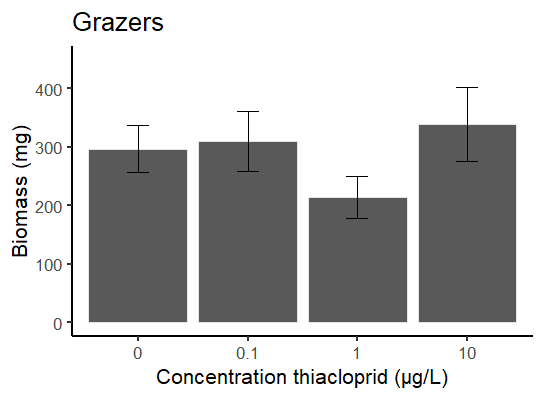

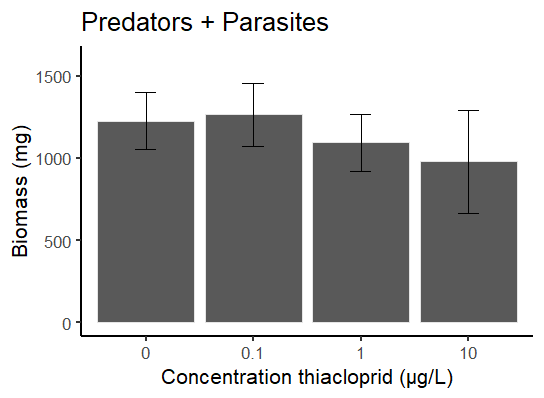
**

**Figure S7.** Average biomass (N = 9) ±SEM per invertebrate functional feeding group per concentration thiacloprid one month after application.

Fig. S8.


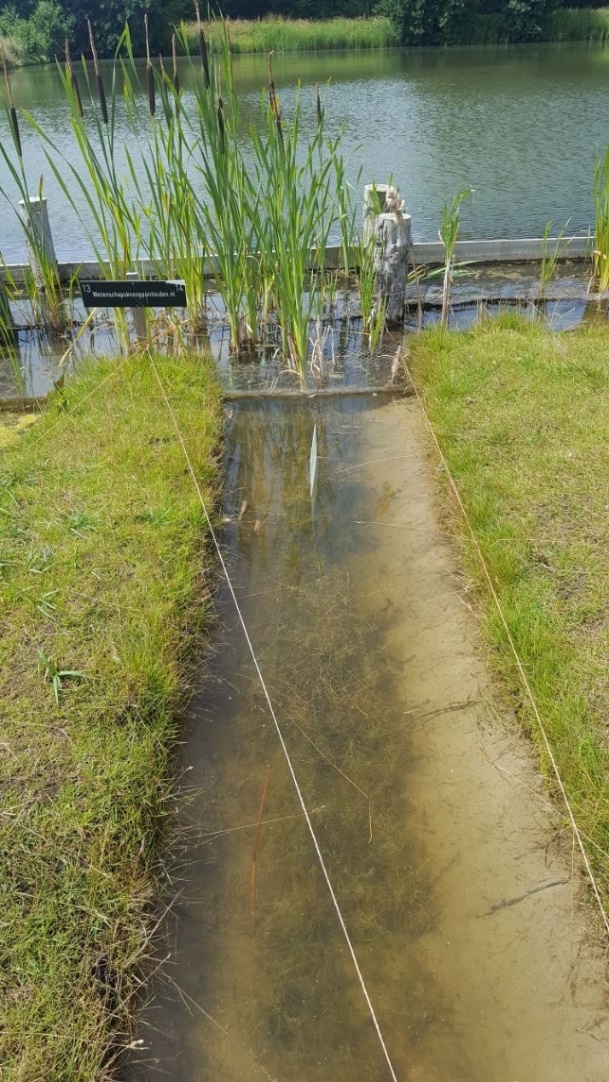

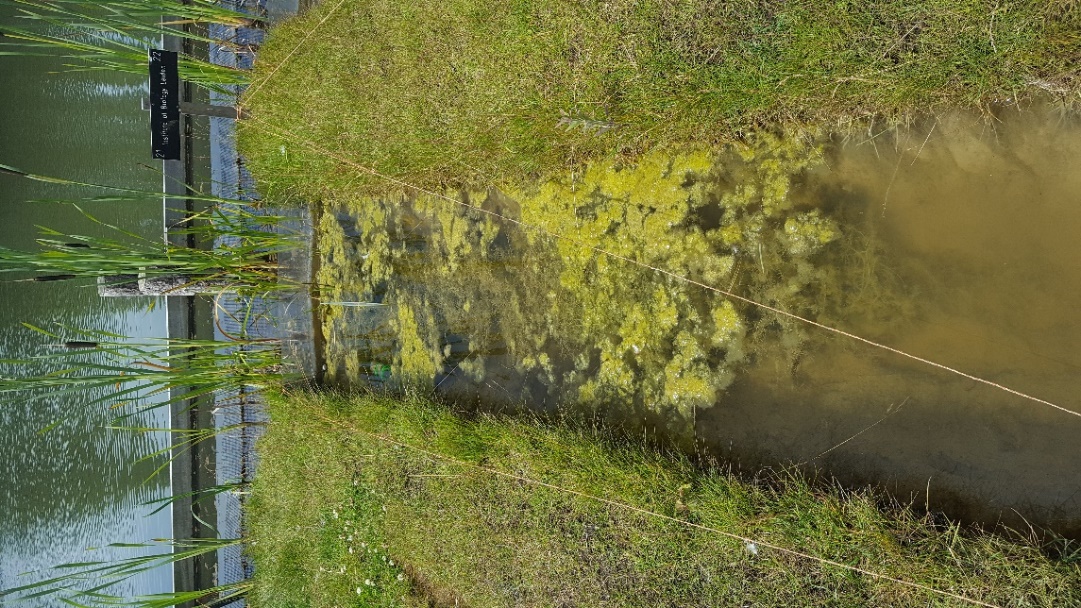


**Figure S8.** Photograph example of the dominance of floating algal beds (FLAB). Left: Control, right: 10µg/L thiacloprid. Photographs were taken one month after the initial thiacloprid spike. FLABs somewhat clumped together dependent on wind conditions (right), but generally spread evenly over the ditch surface.


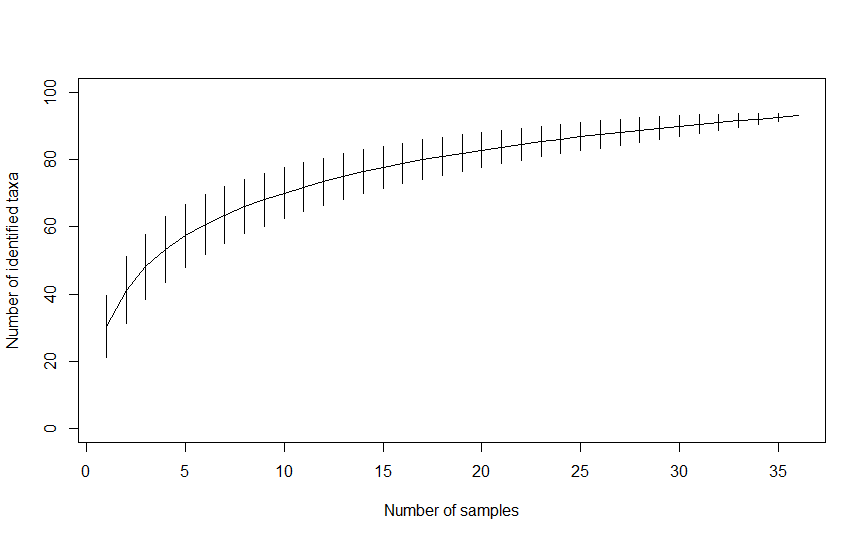
Fig. S9.

**A**


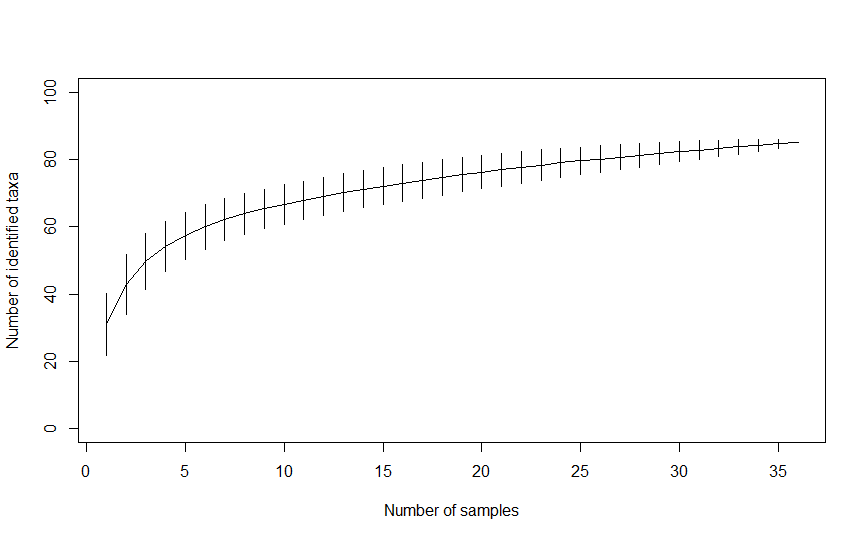


**B**


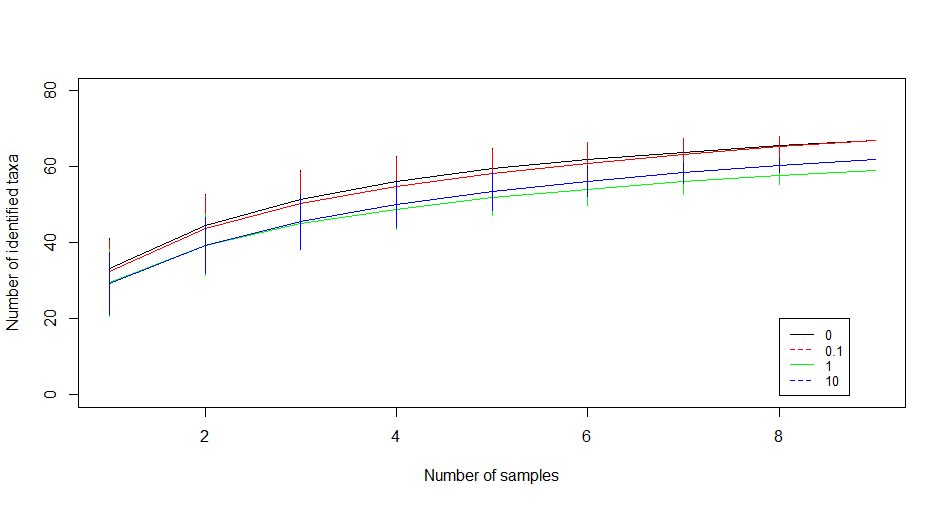


**C**

**Figure S9.** Species accumulation curve (±SD; 999 permutations) of the macroinvertebrate communities (A) one month before (N = 36), one month after the initial thiacloprid spike for (B) all ditches (N = 36) and (C) per treatment (N = 9). Flattening of the curves indicates that the full range of macroinvertebrates (γ-diversity) had been caught.

**References**

Barmentlo, S.H., Parmentier, E.M., de Snoo, G.R. & Vijver, M.G. (2018). Thiacloprid-induced toxicity influenced by nutrients: Evidence from in situ bioassays in experimental ditches. *Environmental Toxicology and Chemistry*, 37.

Barmentlo, S.H., Schrama, M., van Bodegom, P.M., de Snoo, G.R., Musters, C.J.M. & Vijver, M.G. (2019a). Neonicotinoids and fertilizers jointly structure naturally assembled freshwater macroinvertebrate communities. *Science of the Total Environment*, 691, 36–44.

Barmentlo, S.H., Schrama, M., De Snoo, G.R., Van Bodegom, P.M., Van Nieuwenhuijzen, A. & Vijver, M.G. (2021). Experimental evidence for neonicotinoid driven decline in aquatic emerging insects. *Proc Natl Acad Sci U S A*, 118.

Barmentlo, S.H., Vriend, L.M., van Grunsven, R.H.A. & Vijver, M.G. (2019b). Environmental levels of neonicotinoids reduce prey consumption, mobility and emergence of the damselfly *Ischnura elegans*. *Journal of Applied Ecology*, 56, 2034–2044.

Cañedo-Argüelles, M. & Rieradevall, M. (2011). Early succession of the macroinvertebrate community in a shallow lake: Response to changes in the habitat condition. *Limnologica*, 41, 363–370.

Casado, J., Brigden, K., Santillo, D. & Johnston, P. (2019). Screening of pesticides and veterinary drugs in small streams in the European Union by liquid chromatography high resolution mass spectrometry. *Science of the Total Environment*, 670, 1204–1225.

Leiden University (CML) & Rijkswaterstaat-WVL. (2018). *Pesticide Atlas, version 2.0*. Available at: www.bestrijdingsmiddelenatlas.nl. Last accessed 22 November 2018.

Hayasaka, D., Korenaga, T., Sánchez-Bayo, F. & Goka, K. (2012). Differences in ecological impacts of systemic insecticides with different physicochemical properties on biocenosis of experimental paddy fields. *Ecotoxicology*, 21, 191–201.

Morrissey, C.A., Mineau, P., Devries, J.H., Sanchez-Bayo, F., Liess, M., Cavallaro, M.C., *et al.* (2015). Neonicotinoid contamination of global surface waters and associated risk to aquatic invertebrates: A review. *Environ Int*, 74, 291–303.

Roessink I., Merga, L.B., Zweers, P.J. & van den Brink, P.J. (2013). The neonicotinoid imidacloprid shows high chronic toxicity to mayfly nymphs. *Environmental Toxicology and Chemistry*, 32, 1096–1100.

Sánchez-Bayo, F. & Goka, K. (2006). Ecological effects of the insecticide imidacloprid and a pollutant from antidandruff shampoo in experimental rice fields. *Environmental Toxicology and Chemistry*, 25, 1677–1687.

Sánchez-Bayo, F., Goka, K. & Hayasaka, D. (2016). Contamination of the aquatic environment with neonicotinoids and its implication for ecosystems. *Front Environ Sci*, 4.

Stehle, S., Bub, S. & Schulz, R. (2018). Compilation and analysis of global surface water concentrations for individual insecticide compounds. *Science of the Total Environment*, 639, 516–525.
